# Early Supported Discharge and Transitional Care Management After Stroke: A Systematic Review and Meta-Analysis

**DOI:** 10.3389/fneur.2022.755316

**Published:** 2022-03-15

**Authors:** Sungju Jee, Minah Jeong, Nam-Jong Paik, Won-Seok Kim, Yong-Il Shin, Sung-Hwa Ko, In Sun Kwon, Bo Mi Choi, Yunsun Jung, Wonkee Chang, Min Kyun Sohn

**Affiliations:** ^1^Department of Rehabilitation Medicine, Chungnam National University Hospital and Chungnam National University College of Medicine, Daejeon, South Korea; ^2^Department of Rehabilitation Medicine, Seoul National University Bundang Hospital, Seoul National University College of Medicine, Seongnam, South Korea; ^3^Department of Rehabilitation Medicine, Pusan National University School of Medicine, Pusan National University Yangsan Hospital, Pusan, South Korea; ^4^Clinical Trials Center, Chungnam National University Hospital, Daejeon, South Korea; ^5^Department of Public Health and Medical Services, Chungnam National University Hospital, Daejeon, South Korea

**Keywords:** cerebrovascular disease, continuity of patient care, transitional care, rehabilitation, early supported discharge (ESD)

## Abstract

**Objective:**

To investigate the available evidence on early supported discharge (ESD) and transitional care (TC) delivery service in patients with cerebrovascular disease.

**Methods:**

A systematic literature search was conducted to collect all available evidence on the use of ESD and TC services. We included cluster-randomized pragmatic trials or randomized controlled trials (RCTs) that recruited patients with stroke or transient ischemic attack to receive either conventional care or any care service intervention that included rehabilitation or support provided by professional medical personnel with the aim of accelerating and supporting home discharge. Relevant data were electronically searched through international databases (Cochrane Library, EMBASE, and PubMed) and incorporated into a summary grid to investigate research outcomes and provide a narrative synthesis. Furthermore, we compared the outcomes in terms of length of hospital stay, patient and caregiver outcomes, and mortality through meta-analysis.

**Results:**

We identified and included a total of 20 publications of various original randomized studies. There were 18 studies conducted in western countries and 2 in eastern countries. The meta-analysis revealed a tendency that ESD or TC could decrease the length of hospital stay more than the usual care [standardized mean difference (SMD) −0.13; 95% confidence interval (CI) −0.31 to 0.04 days; *P* = 0.14]. Moreover, there was a tendency that ESD resulted in better activities of daily living (ADL) than usual care (SMD 0.29; 95% CI −0.04 to 0.61; *P* = 0.08). Patient outcome based on modified Rankin scale (mRS) score (SMD −0.11; 95% CI −0.38 to 0.17; *P* = 0.45] and mortality (odds ratio 0.80; 95% CI 0.56–1.17; *P* = 0.25) did not reveal any significant difference. The Caregiver Strain Index revealed no difference.

**Conclusion:**

We did not find a large effect size for the use of TC and ESD. When implementing the TC and ESD model from western to Asian countries, services should be prepared and implemented in accordance with national medical rehabilitation pathways for cerebrovascular disease.

## Introduction

In national stroke guidelines of the USA (1), Canada (2), and Scotland (3), early supported discharge (ESD) is recommended as a rehabilitation strategy for post-acute care. In the UK, ESD is part of the stroke care system, and the target group, purpose, scope, and method of ESD are specified in the manuals (4, 5). Langhorne et al. reported that an ESD service comprising a multi-disciplinary team reduced long-term functional dependence and readmission in stroke patients and significantly shortened the length of hospital stay compared with the previous service (6). In particular, the total average length of hospital stay was reduced to 6 days, and the incidence of negative outcomes, such as death or readmission, was reduced by ~5%. No significant differences were noted in the results reported in previous studies; however, the cost of the ESD program was 15–23% lower than that of conventional treatment (6).

In the USA, transitional care (TC) services are provided for various acute diseases (7). TC in the USA must satisfy the following three criteria: contact with the patient within 2 days of discharge, face-to-face follow-up interview/evaluation within 7 or 14 days of discharge depending on the severity of the disease, and non-face-to-face care service according to the patient's needs.

However, in a recent survey on TC including 40 hospitals in North Carolina, only 31.7% of the hospitals satisfied the above-mentioned three criteria of TC (8). Duncan et al. analyzed the effects of nationwide systematic TC in the USA; however, the effects were uncertain (9). The Cochrane review categorized several stages of ESD from full service with mobile rehab team to some minimal counseling before discharge (6). We thought that both ESD and TC could be in the same category of post-acute care (PAC) of stroke with a wide range of spectrum.

In Korea, as the number of cerebrovascular disease patients is increasing due to population aging, efforts are being made to provide standardized acute treatments by developing guidelines for stroke treatment, implementing quality evaluation control by the Health Insurance Review Assessment Service, and opening 14 regional cardio-cerebrovascular centers nationwide. As a result, the mortality rate of acute stroke is decreasing. However, unlike cardiovascular disease, stroke causes neurological disorder. Therefore, the number of stroke survivors who have disabilities after initial treatment and require care in hospitals, facilities, and communities has increased. Accordingly, the medical cost for rehabilitation and management after acute stroke treatment is also increasing. Therefore, there is a need for a continuous management model based on disability and patient characteristics after acute stroke treatment. Studies must focus on developing and standardizing continuous TC and a management model according to the triage for post-stroke care and analyze the effects and hindrance factors of the model in clinical settings.

Therefore, this aimed to investigate the effects of ESD and TC programs on mortality, readmission rate, length of hospital stay, and function through a systematic literature review and meta-analysis of existing and most recent data.

## Materials and Methods

### Data Searches and Sources

A literature search was conducted using databases, including PubMed, EMBASE, and the Cochrane Library. For an extensive literature search, only the participants (P) and intervention (I) were considered, and searches were conducted using keywords, such as “stroke,” “transient ischemic attack (TIA),” “early supported discharge,” “transitional care,” and “rehabilitation.” The databases and search formulas used in each database are presented in Table 1.

**Table 1:** Search strategy according to the searching engine and queries.

	**No**	**Search queries**	**Results (2020.7.7)**
PubMed	#1	“Cerebrovascular Disorders”[Mesh:NoExp]	46,358
	#2	“Cerebrovascular Disorders”[TW] OR “Cerebrovascular Disorder”[TW] OR “Vascular Diseases, Intracranial”[TW] OR “Intracranial Vascular Disease”[TW] OR “Intracranial Vascular Diseases”[TW] OR “Vascular Disease, Intracranial”[TW] OR “Intracranial Vascular Disorders”[TW] OR “Intracranial Vascular Disorder”[TW] OR “Vascular Disorder, Intracranial”[TW] OR “Vascular Disorders, Intracranial”[TW] OR “Cerebrovascular Diseases”[TW] OR “Cerebrovascular Disease”[TW] OR “Disease, Cerebrovascular”[TW] OR “Diseases, Cerebrovascular”[TW] OR “Brain Vascular Disorders”[TW] OR “Brain Vascular Disorder”[TW] OR “Vascular Disorder, Brain”[TW] OR “Vascular Disorders, Brain”[TW] OR “Cerebrovascular Occlusion”[TW] OR “Cerebrovascular Occlusions”[TW] OR “Occlusion, Cerebrovascular”[TW] OR “Occlusions, Cerebrovascular”[TW] OR “Cerebrovascular Insufficiency”[TW] OR “Cerebrovascular Insufficiencies”[TW] OR “Insufficiencies, Cerebrovascular”[TW] OR “Insufficiency, Cerebrovascular”[TW]	63,453
	#3	“Basal Ganglia Cerebrovascular Disease”[MeSH]	558
	#4	“Basal Ganglia Cerebrovascular Disease”[TW] OR “Basal Ganglia Cerebrovascular Disease”[TW] OR “Vascular Diseases, Basal Ganglia”[TW] OR “Vascular Disease, Basal Ganglia”[TW] OR “Basal Ganglia Vascular Disease”[TW] OR “Cerebrovascular Disease, Basal Ganglia”[TW] OR “Lenticulostriate Vasculopathy”[TW] OR “Lenticulostriate Vasculopathies”[TW] OR “Vasculopathies, Lenticulostriate”[TW] OR “Vasculopathy, Lenticulostriate”[TW] OR “Lenticulostriate Vascular Diseases”[TW] OR “Lenticulostriate Vascular Disease”[TW] OR “Vascular Disease, Lenticulostriate”[TW] OR “Vascular Diseases, Lenticulostriate”[TW] OR “Lenticulostriate Diseases, Vascular”[TW] OR “Vascular Lenticulostriate Diseases”[TW]	272
	#5	“Brain Ischemia”[Mesh]	108,434
	#6	“Brain Ischemia”[TW] OR “Brain Ischemias”[TW] OR “Ischemia, Brain”[TW] OR “Ischemic encephalopathy”[TW] OR “Encephalopathy, Ischemic”[TW] OR “Ischemic Encephalopathies”[TW] OR “Cerebral Ischemia”[TW] OR “Cerebral Ischemias”[TW] OR “Ischemias, Cerebral”[TW] OR “Ischemia, Cerebral”[TW]	70,311
	#7	“Cerebral Small Vessel Diseases”[Mesh]	7,467
	#8	“Cerebral Small Vessel Diseases”[TW] OR “Cerebral Small Vessel Disease”[TW] OR “Cerebral Microangiopathies”[TW] OR “Cerebral Microangiopathy”[TW] OR “Microangiopathies, Cerebral”[TW] OR “Microangiopathy, Cerebral”[TW]	2,019
	#9	“Intracranial Arterial Diseases”[Mesh]	62,855
	#10	“Intracranial Arterial Diseases”[TW] OR “Arterial Disease, Intracranial”[TW] OR “Intracranial Arterial Disease”[TW] OR “Intracranial Arterial Disorders”[TW] OR “Arterial Disorder, Intracranial”[TW] OR “Arterial Disorders, Intracranial”[TW] OR “Intracranial Arterial Disorder”[TW] OR “Arterial Diseases, Intracranial”[TW] OR “Brain Diseases, Arterial”[TW] OR “Arterial Brain Disease”[TW] OR “Arterial Diseases, Brain”[TW] OR “Arterial Disease, Brain”[TW] OR “Brain Arterial Disease”[TW] OR “Brain Arterial Diseases”[TW] OR “Brain Disorders, Arterial”[TW] OR “Arterial Brain Disorder”[TW] OR “Arterial Brain Disorders”[TW] OR “Brain Disorder, Arterial”[TW] OR “Arterial Brain Diseases”[TW]	390
	#11	“Intracranial Embolism and Thrombosis”[Mesh]	21,176
	#12	“Intracranial Embolism and Thrombosis”[TW] OR “Cerebral Embolism and Thrombosis”[TW] OR “Brain Embolism and Thrombosis”[TW] OR “Embolism and Thrombosis, Brain”[TW]	8,697
	#13	“Intracranial Hemorrhages”[Mesh]	70,979
	#14	“Intracranial Hemorrhages”[TW] OR “Hemorrhages, Intracranial”[TW] OR “Intracranial Hemorrhage”[TW] OR “Hemorrhage, Intracranial”[TW] OR “Posterior Fossa Hemorrhage”[TW] OR “Hemorrhage, Posterior Fossa”[TW] OR “Hemorrhages, Posterior Fossa”[TW] OR “Posterior Fossa Hemorrhages”[TW] OR “Brain Hemorrhage”[TW] OR “Brain Hemorrhages”[TW] OR “Hemorrhage, Brain”[TW] OR “Hemorrhages, Brain”[TW]	16,875
	#15	“Stroke”[Mesh]	134,064
	#16	“Stroke”[TW] OR “Strokes”[TW] OR “Cerebrovascular Accident”[TW] OR “Cerebrovascular Accidents”[TW] OR “CVA”[TW] OR “CVAs”[TW] OR “Cerebrovascular Apoplexy”[TW] OR “Apoplexy, Cerebrovascular”[TW] OR “Vascular Accident, Brain”[TW] OR “Brain Vascular Accident”[TW] OR “Brain Vascular Accidents”[TW] OR “Vascular Accidents, Brain”[TW] OR “Cerebrovascular Stroke”[TW] OR “Cerebrovascular Strokes”[TW] OR “Stroke, Cerebrovascular”[TW] OR “Strokes, Cerebrovascular”[TW] OR “Apoplexy”[TW] OR “Cerebral Stroke”[TW] OR “Cerebral Strokes”[TW] OR “Stroke, Cerebral”[TW] OR “Strokes, Cerebral”[TW] OR “Stroke, Acute”[TW] OR “Acute Stroke”[TW] OR “Acute Strokes”[TW] OR “Strokes, Acute”[TW] OR “Cerebrovascular Accident, Acute”[TW] OR “Acute Cerebrovascular Accident”[TW] OR “Acute Cerebrovascular Accidents”[TW] OR “Cerebrovascular Accidents, Acute”[TW]	306,011
	#17	#1 OR #2 OR #3 OR #4 OR #5 OR #6 OR #7 OR #8 OR #9 OR #10 OR #11 OR #12 OR #13 OR #14 OR #15 OR #16	508,866
	#18	“Aftercare”[Mesh]	191,753
	#19	“Aftercare”[TW] OR “After Care”[TW] OR “After-Treatment”[TW] OR “After Treatment”[TW] OR “After-Treatments”[TW] OR “Follow-Up Care”[TW] OR “Care, Follow-Up”[TW] OR “Cares, Follow-Up”[TW] OR “Follow Up Care”[TW] OR “Follow-Up Cares”[TW] OR “Postabortion”[TW] OR “Postabortal Programs”[TW] OR “Postabortal Program”[TW] OR “Program, Postabortal”[TW] OR “Programs, Postabortal”[TW]	189,166
	#20	Ambulatory Care“[Mesh]	52,812
	#21	Ambulatory Care”[TW] OR “Care, Ambulatory”[TW] OR “Outpatient Care”[TW] OR “Care, Outpatient”[TW] OR “Health Services, Outpatient”[TW] OR “Health Service, Outpatient”[TW] OR “Outpatient Health Service”[TW] OR “Service, Outpatient Health”[TW] OR “Outpatient Health Services”[TW] OR “Outpatient Services”[TW] OR “Outpatient Service”[TW] OR “Service, Outpatient”[TW] OR “Services, Outpatient”[TW] OR “Services, Outpatient Health”[TW] OR “Urgent Care”[TW] OR “Care, Urgent”[TW] OR “Cares, Urgent”[TW] OR “Urgent Cares”[TW] OR “Clinic Visits”[TW] OR “Clinic Visit”[TW] OR “Visit, Clinic”[TW] OR “Visits, Clinic”[TW]	84,040
	#22	Patient Discharge“[Mesh]	29,666
	#23	Patient Discharge”[TW] OR “Discharge, Patient”[TW] OR “Discharges, Patient”[TW] OR “Patient Discharges”[TW] OR “Discharge Planning”[TW] OR “Discharge Plannings”[TW] OR “Planning, Discharge”[TW] OR “Plannings, Discharge”[TW]	32,631
	#24	Transitional Care“[Mesh]	751
	#25	Transitional Care”[TW] OR “Care, Transitional”[TW] OR “Cares, Transitional”[TW] OR “Transitional Cares”[TW] OR “Transition Care”[TW] OR “Transition Cares”[TW]	2,108
	#26	Stroke Rehabilitation“[Mesh]	13,215
	#27	Stroke Rehabilitation”[TW] OR “Rehabilitation, Stroke”[TW]	14,786
	#28	Home Care Services“[Mesh]	47,203
	#29	Home Care Services”[TW] OR “Home Care Service”[TW] OR “Service, Home Care”[TW] OR “Care Services, Home”[TW] OR “Domiciliary Care”[TW] OR “Care, Domiciliary”[TW] OR “Services, Home Care”[TW] OR “Home Care”[TW] OR “Care, Home”[TW]	52,283
	#30	Progressive Patient Care“[Mesh]	1,202
	#31	Progressive Patient Care”[TW] OR “Care, Progressive Patient”[TW] OR “Cares, Progressive Patient”[TW] OR “Patient Care, Progressive”[TW] OR “Patient Cares, Progressive”[TW] OR “Progressive Patient Cares”[TW]	1,220
	#31	#18 OR #19 OR #20 OR #21 OR #22 OR #23 OR #24 OR #25 OR #26 OR #27 OR #28 OR #29 OR #30 OR #31	545,458
	#32	#17 AND #32	28,121
	**#33**	**#32 Filters: Pragmatic Clinical Trial, Randomized Controlled Trial, from 1997 - 2020**	**3,524**
Cochrane library	#1	MeSH descriptor: “Cerebrovascular Disorders” this term only	1,425
	#2	Cerebrovascular Disorders“:ti, ab, kw OR ”Cerebrovascular Disorder“:ti, ab, kw OR ”Vascular Diseases, Intracranial“:ti ,ab, kw OR ”Intracranial Vascular Disease“:ti, ab, kw OR ”Intracranial Vascular Diseases“:ti, ab, kw OR ”Vascular Disease, Intracranial“:ti, ab, kw OR ”Intracranial Vascular Disorders“:ti, ab, kw OR ”Intracranial Vascular Disorder“:ti, ab, kw OR ”Vascular Disorder, Intracranial“:ti, ab, kw OR ”Vascular Disorders, Intracranial“:ti, ab, kw OR ”Cerebrovascular Diseases“:ti, ab, kw OR ”Cerebrovascular Disease“:ti, ab, kw OR ”Disease, Cerebrovascular“:ti, ab, kw OR ”Diseases, Cerebrovascular“:ti, ab, kw OR ”Brain Vascular Disorders“:ti, ab, kw OR ”Brain Vascular Disorder“:ti, ab, kw OR ”Vascular Disorder, Brain“:ti, ab, kw OR ”Vascular Disorders, Brain“:ti, ab, kw OR ”Cerebrovascular Occlusion“:ti, ab, kw OR ”Cerebrovascular Occlusions“:ti, ab, kw OR ”Occlusion, Cerebrovascular“:ti, ab, kw OR ”Occlusions, Cerebrovascular“:ti, ab, kw OR ”Cerebrovascular Insufficiency“:ti, ab, kw OR ”Cerebrovascular Insufficiencies“:ti, ab, kw OR ”Insufficiencies, Cerebrovascular“:ti, ab, kw OR ”Insufficiency, Cerebrovascular“:ti, ab, kw	4,373
	#3	[mh ”Basal Ganglia Cerebrovascular Disease“]	28
	#4	Basal Ganglia Cerebrovascular Disease”:ti, ab, kw OR “Basal Ganglia Cerebrovascular Disease”:ti, ab, kw OR “Vascular Diseases, Basal Ganglia”:ti, ab, kw OR “Vascular Disease, Basal Ganglia”:ti, ab, kw OR “Basal Ganglia Vascular Disease”:ti, ab, kw OR “Cerebrovascular Disease, Basal Ganglia”:ti, ab, kw OR “Lenticulostriate Vasculopathy”:ti, ab, kw OR “Lenticulostriate Vasculopathies”:ti, ab, kw OR “Vasculopathies, Lenticulostriate”:ti, ab, kw OR “Vasculopathy, Lenticulostriate”:ti, ab, kw OR “Lenticulostriate Vascular Diseases”:ti, ab, kw OR “Lenticulostriate Vascular Disease”:ti, ab, kw OR “Vascular Disease, Lenticulostriate”:ti, ab, kw OR “Vascular Diseases, Lenticulostriate”:ti, ab, kw OR “Lenticulostriate Diseases, Vascular”:ti, ab, kw OR “Vascular Lenticulostriate Diseases”:ti, ab, kw	10
	#5	[mh “Brain Ischemia”]	3,534
	#6	Brain Ischemia“:ti, ab, kw OR ”Brain Ischemias“:ti, ab, kw OR ”Ischemia, Brain“:ti, ab, kw OR ”Ischemic encephalopathy“:ti, ab, kw OR ”Encephalopathy, Ischemic“:ti, ab, kw OR ”Ischemic Encephalopathies“:ti, ab, kw OR ”Cerebral Ischemia“:ti, ab, kw OR ”Cerebral Ischemias“:ti, ab, kw OR ”Ischemias, Cerebral“:ti, ab, kw OR ”Ischemia, Cerebral“:ti, ab, kw	6,452
	#7	[mh ”Cerebral Small Vessel Diseases“]	206
	#8	Cerebral Small Vessel Diseases”:ti, ab, kw OR “Cerebral Small Vessel Disease”:ti, ab, kw OR “Cerebral Microangiopathies”:ti, ab, kw OR “Cerebral Microangiopathy”:ti, ab, kw OR “Microangiopathies, Cerebral”:ti, ab, kw OR “Microangiopathy, Cerebral”:ti, ab, kw	141
	#9	[mh “Intracranial Arterial Diseases”]	1,140
	#10	Intracranial Arterial Diseases“:ti, ab, kw OR ”Arterial Disease, Intracranial“:ti, ab, kw OR ”Intracranial Arterial Disease“:ti, ab, kw OR ”Intracranial Arterial Disorders“:ti, ab, kw OR ”Arterial Disorder, Intracranial“:ti, ab, kw OR ”Arterial Disorders, Intracranial“:ti, ab, kw OR ”Intracranial Arterial Disorder“:ti, ab, kw OR ”Arterial Diseases, Intracranial“:ti, ab, kw OR ”Brain Diseases, Arterial“:ti, ab, kw OR ”Arterial Brain Disease“:ti, ab, kw OR ”Arterial Diseases, Brain“:ti, ab, kw OR ”Arterial Disease, Brain“:ti, ab, kw OR ”Brain Arterial Disease“:ti, ab, kw OR ”Brain Arterial Diseases“:ti, ab, kw OR ”Brain Disorders, Arterial“:ti, ab, kw OR ”Arterial Brain Disorder“:ti, ab, kw OR ”Arterial Brain Disorders“:ti, ab, kw OR ”Brain Disorder, Arterial“:ti, ab, kw OR ”Arterial Brain Diseases“:ti, ab, kw	13
	#11	[mh ”Intracranial Embolism and Thrombosis“]	308
	#12	Intracranial Embolism and Thrombosis”:ti, ab, kw OR “Cerebral Embolism and Thrombosis”:ti, ab, kw OR “Brain Embolism and Thrombosis”:ti, ab, kw OR “Embolism and Thrombosis, Brain”:ti, ab, kw	88
	#13	[mh “Intracranial Hemorrhages”]	1,926
	#14	Intracranial Hemorrhages“:ti, ab, kw OR ”Hemorrhages, Intracranial“:ti, ab, kw OR ”Intracranial Hemorrhage“:ti, ab, kw OR ”Hemorrhage, Intracranial“:ti, ab, kw OR ”Posterior Fossa Hemorrhage“:ti, ab, kw OR ”Hemorrhage, Posterior Fossa“:ti, ab, kw OR ”Hemorrhages, Posterior Fossa“:ti, ab, kw OR ”Posterior Fossa Hemorrhages“:ti, ab, kw OR ”Brain Hemorrhage“:ti, ab, kw OR ”Brain Hemorrhages“:ti, ab, kw OR ”Hemorrhage, Brain“:ti, ab, kw OR ”Hemorrhages, Brain“:ti, ab, kw	4,678
	#15	[mh ”Stroke“]	9,502
	#16	Stroke”:ti, ab, kw OR “Strokes”:ti, ab, kw OR “Cerebrovascular Accident”:ti, ab, kw OR “Cerebrovascular Accidents”:ti, ab, kw OR “CVA”:ti, ab, kw OR “CVAs”:ti, ab, kw OR “Cerebrovascular Apoplexy”:ti, ab, kw OR “Apoplexy, Cerebrovascular”:ti, ab, kw OR “Vascular Accident, Brain”:ti, ab, kw OR “Brain Vascular Accident”:ti, ab, kw OR “Brain Vascular Accidents”:ti, ab, kw OR “Vascular Accidents, Brain”:ti, ab, kw OR “Cerebrovascular Stroke”:ti, ab, kw OR “Cerebrovascular Strokes”:ti, ab, kw OR “Stroke, Cerebrovascular”:ti, ab, kw OR “Strokes, Cerebrovascular”:ti, ab, kw OR “Apoplexy”:ti, ab, kw OR “Cerebral Stroke”:ti, ab, kw OR “Cerebral Strokes”:ti, ab, kw OR “Stroke, Cerebral”:ti, ab, kw OR “Strokes, Cerebral”:ti, ab, kw OR “Stroke, Acute”:ti, ab, kw OR “Acute Stroke”:ti, ab, kw OR “Acute Strokes”:ti, ab, kw OR “Strokes, Acute”:ti, ab, kw OR “Cerebrovascular Accident, Acute”:ti, ab, kw OR “Acute Cerebrovascular Accident”:ti, ab, kw OR “Acute Cerebrovascular Accidents”:ti, ab, kw OR “Cerebrovascular Accidents, Acute”:ti, ab, kw	57,080
	#17	#1 OR #2 OR #3 OR #4 OR #5 OR #6 OR #7 OR #8 OR #9 OR #10 OR #11 OR #12 OR #13 OR #14 OR #15 OR #16	64,896
	#18	[mh “Aftercare”]	22,451
	#19	Aftercare“:ti, ab, kw OR ”After Care“:ti, ab, kw OR ”After-Treatment“:ti, ab, kw OR ”After Treatment“:ti, ab, kw OR ”After-Treatments“:ti, ab, kw OR ”Follow-Up Care“:ti, ab, kw OR ”Care, Follow-Up“:ti, ab, kw OR ”Cares, Follow-Up“:ti, ab, kw OR ”Follow Up Care“:ti, ab, kw OR ”Follow-Up Cares“:ti, ab, kw OR ”Postabortion“:ti, ab, kw OR ”Postabortal Programs“:ti, ab, kw OR ”Postabortal Program“:ti, ab, kw OR ”Program, Postabortal“:ti, ab, kw OR ”Programs, Postabortal“:ti, ab, kw	44,805
	#20	[mh ”Ambulatory Care“]	3,605
	#21	Ambulatory Care”:ti, ab, kw OR “Care, Ambulatory”:ti, ab, kw OR “Outpatient Care”:ti, ab, kw OR “Care, Outpatient”:ti, ab, kw OR “Health Services, Outpatient”:ti, ab, kw OR “Health Service, Outpatient”:ti, ab, kw OR “Outpatient Health Service”:ti, ab, kw OR “Service, Outpatient Health”:ti, ab, kw OR “Outpatient Health Services”:ti, ab, kw OR “Outpatient Services”:ti, ab, kw OR “Outpatient Service”:ti, ab, kw OR “Service, Outpatient”:ti, ab, kw OR “Services, Outpatient”:ti, ab, kw OR “Services, Outpatient Health”:ti, ab, kw OR “Urgent Care”:ti, ab, kw OR “Care, Urgent”:ti, ab, kw OR “Cares, Urgent”:ti, ab, kw OR “Urgent Cares”:ti, ab, kw OR “Clinic Visits”:ti, ab, kw OR “Clinic Visit”:ti, ab, kw OR “Visit, Clinic”:ti, ab, kw OR “Visits, Clinic”:ti, ab, kw	11,153
	#22	[mh “Patient Discharge”]	1,444
	#23	Patient Discharge“:ti, ab, kw OR ”Discharge, Patient“:ti, ab, kw OR ”Discharges, Patient“:ti, ab, kw OR ”Patient Discharges“:ti, ab, kw OR ”Discharge Planning“:ti, ab, kw OR ”Discharge Plannings“:ti, ab, kw OR ”Planning, Discharge“:ti, ab, kw OR ”Plannings, Discharge“:ti, ab, kw	2,325
	#24	[mh ”Transitional Care“]	52
	#25	Transitional Care”:ti, ab, kw OR “Care, Transitional”:ti, ab, kw OR “Cares, Transitional”:ti, ab, kw OR “Transitional Cares”:ti, ab, kw OR “Transition Care”:ti, ab, kw OR “Transition Cares”:ti, ab, kw	428
	#26	[mh “Stroke Rehabilitation”]	2,378
	#27	Stroke Rehabilitation“:ti, ab, kw OR ”Rehabilitation, Stroke“:ti, ab, kw	3,807
	#28	[mh ”Home Care Services“]	2,395
	#29	Home Care Services”:ti, ab, kw OR “Home Care Service”:ti, ab, kw OR “Service, Home Care”:ti, ab, kw OR “Care Services, Home”:ti, ab, kw OR “Domiciliary Care”:ti, ab, kw OR “Care, Domiciliary”:ti, ab, kw OR “Services, Home Care”:ti, ab, kw OR “Home Care”:ti, ab, kw OR “Care, Home”:ti, ab, kw	5,461
	#30	[mh “Progressive Patient Care”]	12
	#31	Progressive Patient Care“:ti, ab, kw OR ”Care, Progressive Patient“:ti, ab, kw OR ”Cares, Progressive Patient“:ti, ab, kw OR ”Patient Care, Progressive“:ti, ab, kw OR ”Patient Cares, Progressive“:ti, ab, w OR ”Progressive Patient Cares":ti, ab, kw	16
	#32	#18 OR #19 OR #20 OR #21 OR #22 OR #23 OR #24 OR #25 OR #26 OR #27 OR #28 OR #29 OR #30 OR #31	84,278
	#33	#17 AND #32	6,937
	**#34**	**Filters: 1997 - 2020**	**6676**
EMBASE (Elsevier)	#1	cerebrovascular disease'/de	63,413
	#2	Cerebrovascular Disorders':ti, ab, kw,de OR 'Cerebrovascular Disorder':ti, ab, kw,de OR 'Vascular Diseases, Intracranial':ti, ab, kw,de OR 'Intracranial Vascular Disease':ti, ab, kw,de OR 'Intracranial Vascular Diseases':ti, ab, kw,de OR 'Vascular Disease, Intracranial':ti, ab, kw,de OR 'Intracranial Vascular Disorders':ti, ab, kw,de OR 'Intracranial Vascular Disorder':ti, ab, kw,de OR 'Vascular Disorder, Intracranial':ti, ab, kw,de OR 'Vascular Disorders, Intracranial':ti, ab, kw,de OR 'Cerebrovascular Diseases':ti, ab, kw,de OR 'Cerebrovascular Disease':ti, ab, kw,de OR 'Disease, Cerebrovascular':ti, ab, kw,de OR 'Diseases, Cerebrovascular':ti, ab, kw,de OR 'Brain Vascular Disorders':ti, ab, kw,de OR 'Brain Vascular Disorder':ti, ab, kw,de OR 'Vascular Disorder, Brain':ti, ab, kw,de OR 'Vascular Disorders, Brain':ti, ab, kw,de OR 'Cerebrovascular Occlusion':ti, ab, kw,de OR 'Cerebrovascular Occlusions':ti, ab, kw,de OR 'Occlusion, Cerebrovascular':ti, ab, kw,de OR 'Occlusions, Cerebrovascular':ti, ab, kw,de OR 'Cerebrovascular Insufficiency':ti, ab, kw,de OR 'Cerebrovascular Insufficiencies':ti, ab, kw,de OR 'Insufficiencies, Cerebrovascular':ti, ab, kw,de OR 'Insufficiency, Cerebrovascular':ti, ab, kw,de	89,473
	#3	basal ganglion hemorrhage'/exp	771
	#4	Basal Ganglia Cerebrovascular Disease':ti, ab, kw,de OR 'Basal Ganglia Cerebrovascular Disease':ti, ab, kw,de OR 'Vascular Diseases, Basal Ganglia':ti, ab, kw,de OR 'Vascular Disease, Basal Ganglia':ti, ab, kw,de OR 'Basal Ganglia Vascular Disease':ti, ab, kw,de OR 'Cerebrovascular Disease, Basal Ganglia':ti, ab, kw,de OR 'Lenticulostriate Vasculopathy':ti, ab, kw,de OR 'Lenticulostriate Vasculopathies':ti, ab, kw,de OR 'Vasculopathies, Lenticulostriate':ti, ab, kw,de OR 'Vasculopathy, Lenticulostriate':ti, ab, kw,de OR 'Lenticulostriate Vascular Diseases':ti, ab, kw,de OR 'Lenticulostriate Vascular Disease':ti, ab, kw,de OR 'Vascular Disease, Lenticulostriate':ti, ab, kw,de OR 'Vascular Diseases, Lenticulostriate':ti, ab, kw,de OR 'Lenticulostriate Diseases, Vascular':ti, ab, kw,de OR 'Vascular Lenticulostriate Diseases':ti, ab, kw,de	108
	#5	brain ischemia'/exp	186,618
	#6	'Brain Ischemia':ti, ab, kw,de OR 'Brain Ischemias':ti, ab, kw,de OR 'Ischemia, Brain':ti, ab, kw,de OR 'Ischemic Encephalopathy':ti, ab, kw,de OR 'Encephalopathy, Ischemic':ti, ab, kw,de OR 'Ischemic Encephalopathies':ti, ab, kw,de OR 'Cerebral Ischemia':ti, ab, kw,de OR 'Cerebral Ischemias':ti, ab, kw,de OR 'Ischemias, Cerebral':ti, ab, kw,de OR 'Ischemia, Cerebral':ti, ab, kw,de OR 'acute ischaemic stroke':ti, ab, kw,de OR 'brain ischemia':ti, ab, kw,de OR 'acute ischemic stroke':ti, ab, kw,de OR 'brain arterial insufficiency':ti, ab, kw,de OR 'brain circulation disorder':ti, ab, kw,de OR 'brain ischaemia':ti, ab, kw,de OR 'cerebral blood circulation disorder':ti, ab, kw,de OR 'cerebral blood flow disorder':ti, ab, kw,de OR 'cerebral circulation disorder':ti, ab, kw,de OR 'cerebral circulatory disorder':ti, ab, kw,de OR 'cerebral ischaemia':ti, ab, kw,de OR 'cerebral ischemia':ti, ab, kw,de OR 'cerebrovascular circulation disorder':ti, ab, kw,de OR 'cerebrovascular ischaemia':ti, ab, kw,de OR 'cerebrovascular ischemia':ti, ab, kw,de OR 'chronic ischaemic stroke':ti, ab, kw,de OR 'chronic ischemic stroke':ti, ab, kw,de OR 'ischaemia cerebri':ti, ab, kw,de OR 'ischaemic brain disease':ti, ab, kw,de OR 'ischaemic encephalopathy':ti, ab, kw,de OR 'ischaemic stroke':ti, ab, kw,de OR 'ischemia cerebri':ti, ab, kw,de OR 'ischemic brain disease':ti, ab, kw,de OR 'ischemic encephalopathy':ti, ab, kw,de OR 'ischemic stroke':ti, ab, kw,de OR 'neural ischaemia':ti, ab, kw,de OR 'neural ischemia':ti, ab, kw,de	183,481
	#7	cerebrovascular disease'/exp	733,323
	#8	Cerebral Small Vessel Diseases':ti, ab, kw,de OR 'Cerebral Small Vessel Disease':ti, ab, kw,de OR 'Cerebral Microangiopathies':ti, ab, kw,de OR 'Cerebral Microangiopathy':ti, ab, kw,de OR 'Microangiopathies, Cerebral':ti, ab, kw,de OR 'Microangiopathy, Cerebral':ti, ab, kw,de OR 'cerebrovascular disease':ti, ab, kw,de OR 'brain angiopathy':ti, ab, kw,de OR 'brain circulation failure':ti, ab, kw,de OR 'brain vascular disease':ti, ab, kw,de OR 'brain vasculopathy':ti, ab, kw,de OR 'cerebral small vessel disease':ti, ab, kw,de OR 'cerebral small vessel diseases':ti, ab, kw,de OR 'cerebral vascular disease':ti, ab, kw,de OR 'cerebral vascular disorder':ti, ab, kw,de OR 'cerebral vascular disturbance':ti, ab, kw,de OR 'cerebral vascular lesion':ti, ab, kw,de OR 'cerebral vasculopathy':ti, ab, kw,de OR 'cerebrovascular damage':ti, ab, kw,de OR 'cerebrovascular disorder':ti, ab, kw,de OR 'cerebrovascular disorders':ti, ab, kw,de OR 'cerebrovascular lesion':ti, ab, kw,de OR 'cerebrovascular pathology':ti, ab, kw,de OR 'cerebrovascular syndrome':ti, ab, kw,de	87,618
	#9	cerebral artery disease'/exp	5,201
	#10	Intracranial Arterial Diseases':ti, ab, kw,de OR 'Arterial Disease, Intracranial':ti, ab, kw,de OR 'Intracranial Arterial Disease':ti, ab, kw,de OR 'Intracranial Arterial Disorders':ti, ab, kw,de OR 'Arterial Disorder, Intracranial':ti, ab, kw,de OR 'Arterial Disorders, Intracranial':ti, ab, kw,de OR 'Intracranial Arterial Disorder':ti, ab, kw,de OR 'Arterial Diseases, Intracranial':ti, ab, kw,de OR 'Brain Diseases, Arterial':ti, ab, kw,de OR 'Arterial Brain Disease':ti, ab, kw,de OR 'Arterial Diseases, Brain':ti, ab, kw,de OR 'Arterial Disease, Brain':ti, ab, kw,de OR 'Brain Arterial Disease':ti, ab, kw,de OR 'Brain Arterial Diseases':ti, ab, kw,de OR 'Brain Disorders, Arterial':ti, ab, kw,de OR 'Arterial Brain Disorder':ti, ab, kw,de OR 'Arterial Brain Disorders':ti, ab, kw,de OR 'Brain Disorder, Arterial':ti, ab, kw,de OR 'Arterial Brain Diseases':ti, ab, kw,de	105
	#11	thromboembolism'/exp	513,919
	#12	Intracranial Embolism and Thrombosis':ti, ab, kw,de OR 'Cerebral Embolism and Thrombosis':ti, ab, kw,de OR 'Brain Embolism and Thrombosis':ti, ab, kw,de OR 'Embolism and Thrombosis, Brain':ti, ab, kw,de	721
	#13	brain hemorrhage'/exp	145,378
	#14	Intracranial Hemorrhages':ti, ab, kw,de OR 'Hemorrhages, Intracranial':ti, ab, kw,de OR 'Intracranial Hemorrhage':ti, ab, kw,de OR 'Hemorrhage, Intracranial':ti, ab, kw,de OR 'Posterior Fossa Hemorrhage':ti, ab, kw,de OR 'Hemorrhage, Posterior Fossa':ti, ab, kw,de OR 'Hemorrhages, Posterior Fossa':ti, ab, kw,de OR 'Posterior Fossa Hemorrhages':ti, ab, kw,de OR 'Brain Hemorrhage':ti, ab, kw,de OR 'Brain Hemorrhages':ti, ab, kw,de OR 'Hemorrhage, Brain':ti, ab, kw,de OR 'Hemorrhages, Brain':ti, ab, kw,de	109,963
	#15	cerebrovascular accident'/exp	327,527
	#16	Stroke':ti, ab, kw,de OR 'Strokes':ti, ab, kw,de OR 'Cerebrovascular Accident':ti, ab, kw,de OR 'Cerebrovascular Accidents':ti, ab, kw,de OR 'CVA':ti, ab, kw,de OR 'CVAs':ti, ab, kw,de OR 'Cerebrovascular Apoplexy':ti, ab, kw,de OR 'Apoplexy, Cerebrovascular':ti, ab, kw,de OR 'Vascular Accident, Brain':ti, ab, kw,de OR 'Brain Vascular Accident':ti, ab, kw,de OR 'Brain Vascular Accidents':ti, ab, kw,de OR 'Vascular Accidents, Brain':ti, ab, kw,de OR 'Cerebrovascular Stroke':ti, ab, kw,de OR 'Cerebrovascular Strokes':ti, ab, kw,de OR 'Stroke, Cerebrovascular':ti, ab, kw,de OR 'Strokes, Cerebrovascular':ti, ab, kw,de OR 'Apoplexy':ti, ab, kw,de OR 'Cerebral Stroke':ti, ab, kw,de OR 'Cerebral Strokes':ti, ab, kw,de OR 'Stroke, Cerebral':ti, ab, kw,de OR 'Strokes, Cerebral':ti, ab, kw,de OR 'Stroke, Acute':ti, ab, kw,de OR 'Acute Stroke':ti, ab, kw,de OR 'Acute Strokes':ti, ab, kw,de OR 'Strokes, Acute':ti, ab, kw,de OR 'Cerebrovascular Accident, Acute':ti, ab, kw,de OR 'Acute Cerebrovascular Accident':ti, ab, kw,de OR 'Acute Cerebrovascular Accidents':ti, ab, kw,de OR 'Cerebrovascular Accidents, Acute':ti, ab, kw,de	513,283
	#17	#1 OR #2 OR #3 OR #4 OR #5 OR #6 OR #7 OR #8 OR #9 OR #10 OR #11 OR #12 OR #13 OR #14 OR #15 OR #16	1,267,908
	#18	aftercare'/exp	1,569,098
	#19	Aftercare':ti, ab, kw,de OR 'After Care':ti, ab, kw,de OR 'After-Treatment':ti, ab, kw,de OR 'After Treatment':ti, ab, kw,de OR 'After-Treatments':ti, ab, kw,de OR 'Follow-Up Care':ti, ab, kw,de OR 'Care, Follow-Up':ti, ab, kw,de OR 'Cares, Follow-Up':ti, ab, kw,de OR 'Follow Up Care':ti, ab, kw,de OR 'Follow-Up Cares':ti, ab, kw,de OR 'Postabortion':ti, ab, kw,de OR 'Postabortal Programs':ti, ab, kw,de OR 'Postabortal Program':ti, ab, kw,de OR 'Program, Postabortal':ti, ab, kw,de OR 'Programs, Postabortal':ti, ab, kw,de	261,420
	#20	ambulatory care'/exp	50,314
	#21	Ambulatory Care':ti, ab, kw,de OR 'Care, Ambulatory':ti, ab, kw,de OR 'Outpatient Care':ti, ab, kw,de OR 'Care, Outpatient':ti, ab, kw,de OR 'Health Services, Outpatient':ti, ab, kw,de OR 'Health Service, Outpatient':ti, ab, kw,de OR 'Outpatient Health Service':ti, ab, kw,de OR 'Service, Outpatient Health':ti, ab, kw,de OR 'Outpatient Health Services':ti, ab, kw,de OR 'Outpatient Services':ti, ab, kw,de OR 'Outpatient Service':ti, ab, kw,de OR 'Service, Outpatient':ti, ab, kw,de OR 'Services, Outpatient':ti, ab, kw,de OR 'Services, Outpatient Health':ti, ab, kw,de OR 'Urgent Care':ti, ab, kw,de OR 'Care, Urgent':ti, ab, kw,de OR 'Cares, Urgent':ti, ab, kw,de OR 'Urgent Cares':ti, ab, kw,de OR 'Clinic Visits':ti, ab, kw,de OR 'Clinic Visit':ti, ab, kw,de OR 'Visit, Clinic':ti, ab, kw,de OR 'Visits, Clinic':ti, ab, kw,de	105,250
	#22	hospital discharge'/exp	124,630
	#23	Patient Discharge':ti, ab, kw,de OR 'Discharge, Patient':ti, ab, kw,de OR 'Discharges, Patient':ti, ab, kw,de OR 'Patient Discharges':ti, ab, kw,de OR 'Discharge Planning':ti, ab, kw,de OR 'Discharge Plannings':ti, ab, kw,de OR 'Planning, Discharge':ti, ab, kw,de OR 'Plannings, Discharge':ti, ab, kw,de	8,399
	#24	transitional care'/exp	2,855
	#25	Transitional Care':ti, ab, kw,de OR 'Care, Transitional':ti, ab, kw,de OR 'Cares, Transitional':ti, ab, kw,de OR 'Transitional Cares':ti, ab, kw,de OR 'Transition Care':ti, ab, kw,de OR 'Transition Cares':ti, ab, kw,de	4,427
	#26	stroke rehabilitation'/exp	3,448
	#27	Stroke Rehabilitation':ti, ab, kw,de OR 'Rehabilitation, Stroke':ti, ab, kw,de	8,316
	#28	home care'/exp	75,309
	#29	Home Care Services':ti, ab, kw,de OR 'Home Care Service':ti, ab, kw,de OR 'Service, Home Care':ti, ab, kw,de OR 'Care Services, Home':ti, ab, kw,de OR 'Domiciliary Care':ti, ab, kw,de OR 'Care, Domiciliary':ti, ab, kw,de OR 'Services, Home Care':ti, ab, kw,de OR 'Home Care':ti, ab, kw,de OR 'Care, Home':ti, ab, kw,de	71,244
	#30	progressive patient care'/exp	996
	#31	Progressive Patient Care':ti, ab, kw,de OR 'Care, Progressive Patient':ti, ab, kw,de OR 'Cares, Progressive Patient':ti, ab, kw,de OR 'Patient Care, Progressive':ti, ab, kw,de OR 'Patient Cares, Progressive':ti, ab, kw,de OR 'Progressive Patient Cares':ti, ab, kw,de	1,079
	#32	#18 OR #19 OR #20 OR #21 OR #22 OR #23 OR #24 OR #25 OR #26 OR #27 OR #28 OR #29 OR #30 OR #31	2,059,138
	#33	#17 AND #32	187,923
	#34	#32 Filters: Randomized Controlled Trial, from 1997 - 2020	10,886

### Study Selection

The key question was selected according to a discussion about previous studies and expert opinion. The key question was “Can transitional care, including early supported discharge (ESD), have an impact on functional outcomes, readmission, mortality, and length of hospital stay after acute cerebrovascular accident?” Table 2 describes the detailed strategies for study inclusion, including population, intervention/comparator, outcomes, time, setting, and design (PICOTS-SD). We included randomized pragmatic trials (RPTs) or randomized controlled trials (RCTs) that recruited patients with stroke or transient ischemic attack to receive either conventional care or any care service intervention, wherein rehabilitation or support was provided by professional medical personnel with the aim of accelerating and supporting home discharge. The RPT provides a real-world assessment of a new care model vs. the usual care. It did not include a control group but a usual care group was examined (9). The outcome parameters for effectiveness included functional status, readmission rate, mortality, and caregiver burden. We selected studies published after 1997, given that there were significant changes in the PAC of stroke patients, such as ESD implementation in London and UK.

**Table 2:** Strategy for study inclusion.

	**Contents**
Population	Stroke or transient ischemic attack (TIA)
Intervention/ comparator	Transitional care or early supported discharge program by hospital professionals/usual care
Outcomes	ADL, mRS, death, Quality of life, readmission rate, total length of hospital stay, care giver burden
Time	·Publication: after 1997 ·Duration of study and follow up: no limitation
Setting	–
Study design	·Randomized controlled clinical trial, RCT ·Randomized pragmatic controlled clinical trial, RPT

### Literature Screening Strategy

**Figure 1:**
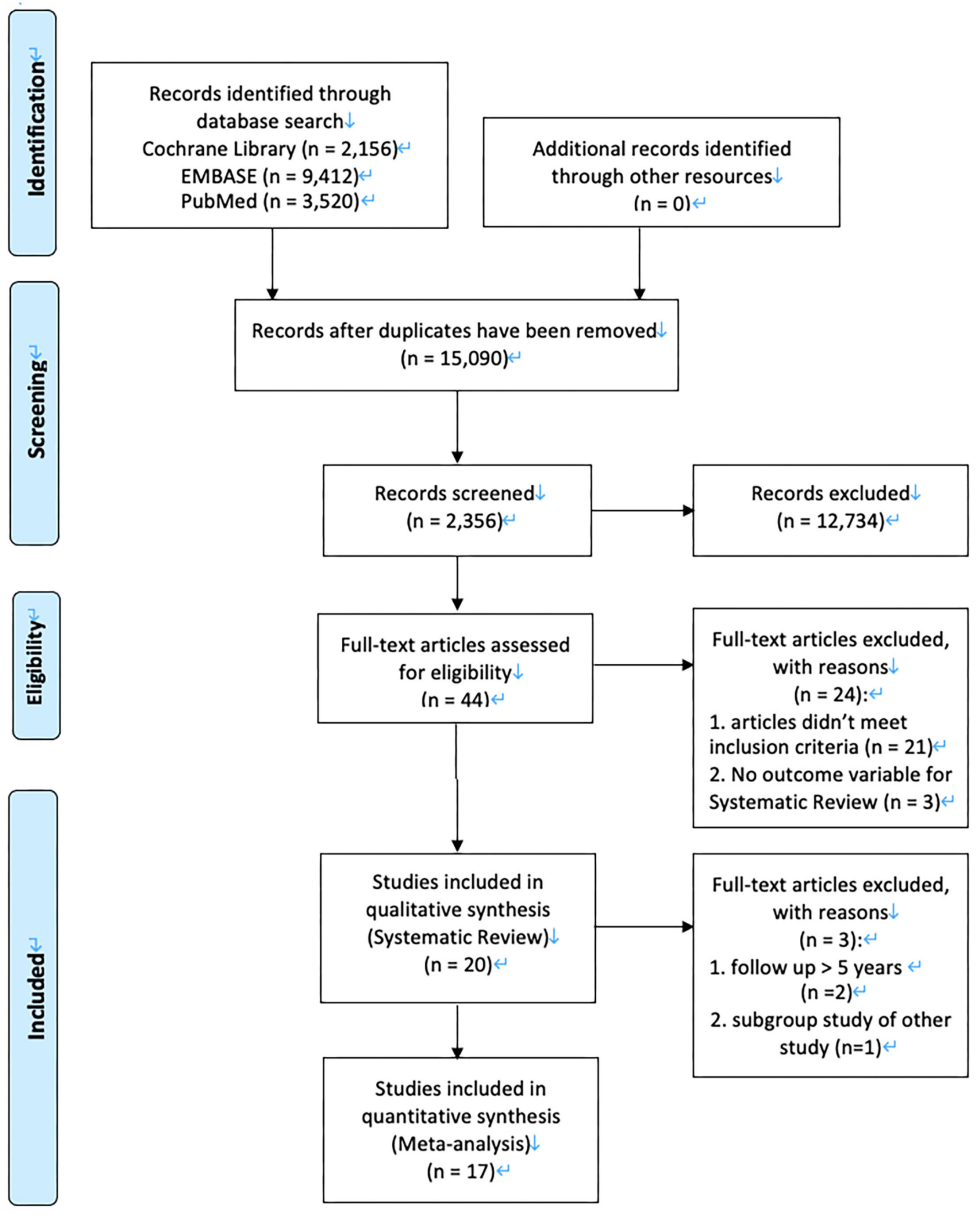
PRISMA flow sheet.

All the articles searched on each database were merged, and articles that were searched multiple times were removed. Thereafter, the title and abstract of the studies were reviewed to exclude irrelevant studies. If no decision could be made based on the title and abstract, the full text was systematically reviewed and analyzed. The literature review and analysis were conducted by the main researcher and another researcher with experience as an occupational therapist. Data were extracted by choosing necessary items from the list of available data in the selected articles. Two or more researchers independently extracted the data, and disagreements were settled through discussion. The main data that were extracted included study characteristics (study design, country, period, and patient inclusion criteria), patient characteristics (number of participants, disease classification, interventions for intervention and control groups, and location), and clinical outcomes. Each variable is described in the Supplementary Table 1. TC interventions were divided into two types: type I for interventions performed by medical staff, including doctors and nurses, excluding visiting rehabilitation; and type II for interventions that included visiting rehabilitation. This was a modified version of the method used in the Cochrane review (6). The key search terms related to “early supported discharge for stroke patients” were used to search articles in international literature search databases such as PubMed, Embase, and the Cochrane Library. The total number of searched articles was 15,090, excluding those that were searched multiple times. The search formulas for each database are shown in the Table 1. In the 1st literature selection process, the title of the studies was reviewed to assess relevance. In the 2nd selection process, the abstract was reviewed to assess relevance to key questions, and a total of 44 studies were selected. Lastly, the main text of articles selected in the 2nd selection process was reviewed according to the selection and exclusion criteria. Finally, 20 articles (articles 1–20) were selected for systematic literature review. In the meta-analysis process, the subgroup study (*n* = 1) of other study and long-term (>5 years) follow-up studies (*n* = 2) were excluded to reduce bias. Figure 1 illustrates a flowchart for selection of the articles included in this systematic literature review. Articles 5, 12, and 19 were excluded in the quantitative analysis process.

### Quality Assessment of the Literature

The risk of bias (RoB) of the studies was assessed using the Cochrane risk-of-bias tool. Two researchers independently assessed the RoB of the selected studies, and disagreements were settled through discussion to reach a consensus. The detailed guidelines are described in Supplementary Table 2. RoB. If there was insufficient information about the relevant items, the RoB was evaluated as “uncertain (yellow).” If the RoB was small, it was evaluated as “low (green).” We measured RoB for only 17 RCTs excluding one RPT (article 9).

### Statistical Analysis

Data were extracted in accordance with several categories, including study characteristics (design, country, duration, inclusion criteria), patient characteristics (numbers, disease categories, type of intervention, age, the composition of case and control group, place, duration of intervention, and frequency), and clinical outcomes. If a quantitative measurement was possible, we conducted a meta-analysis and confirmed heterogeneity, or we described the data qualitatively. We used the outcome value at the longest follow-up of each study. Dichotomous outcomes are presented as relative risks with 95% confidence intervals (CIs). Variance and heterogeneity among the included studies were explored using forest plots and I^2^ statistics, respectively. Data from each study were pooled using a fixed-effects meta-analysis model for the analysis with I^2^ > 75% as well as by the random-effects model. If statistical heterogeneity was identified, meta-regression was conducted to explore the covariance, which affects the random effects, and to confirm the reason for heterogeneity. All statistical analyses were performed using the Review Manager 5.3 software (RevMan 2014, The Nordic Cochrane Centre, Copenhagen, Denmark).

## Results

### Systematic Review

#### General Characteristics and Various Outcomes of Included Studies

This literature review finally examined 20 articles, of which 19 were RCTs and 1 was an RPT. Regarding international location, 85% of the studies were conducted in Europe [Norway (10–15): 6, Denmark (16–20): 5, Netherlands (21): 1, Sweden (22, 23): 2, UK (24, 25): 2, Portugal (26): 1], 10% were conducted in Asia [China (27): 1, Hong Kong (28): 1], and 5% were conducted in North America [USA (9): 1]. For the intervention types, seven studies provided type I intervention, whereas 13 studies provided type II intervention. The greatest number of studies (38%) followed up the participants for more than 12 months, whereas 33% and 28% of the studies followed up the participants for 3–6 months and <3 months, respectively. The publication year ranged from 1997 to June 2020. The greatest number of studies was published in 2004 (four studies). Two studies were published in each of 2002, 2019, and 2020, whereas one study was published each in 1997, 2000, 2001, 2003, 2005, 2009, 2011, 2014, 2015, 2016, and 2017. The list of the selected studies and the characteristics of each study are shown in Supplementary Table 3. The study design, study country, and publication year were described in a reverse order. The outcomes (measured values) of each study are summarized in Supplementary Table 4.

### Meta-Analysis

#### Patient Outcomes

##### Length of Hospital Stay

**Figure 2:**
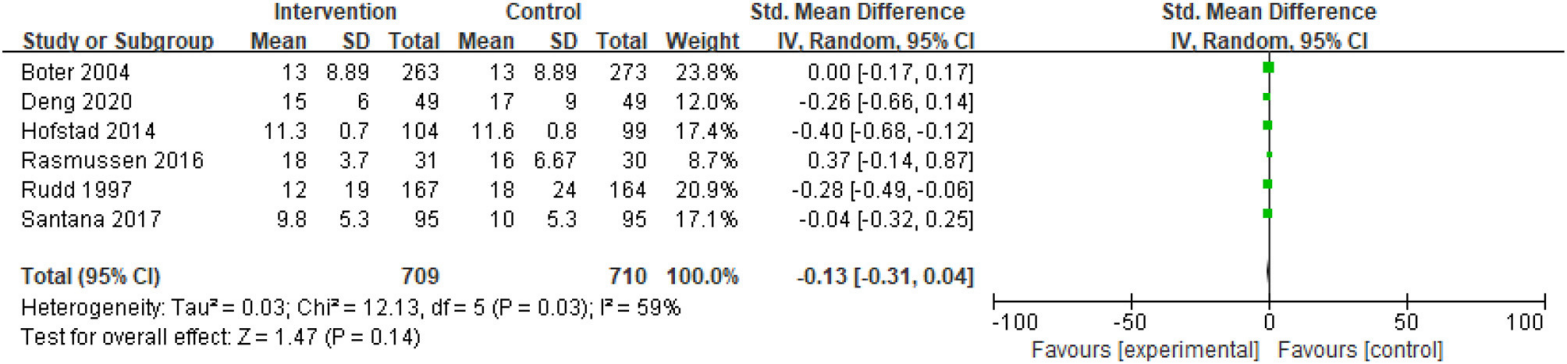
Comparison ESD care vs. usual care; outcome I: length of hospital stay.

The length of hospital stay was reported in six studies. A meta-analysis of the length of hospital stays using the fixed effect model revealed significant heterogeneity among the studies (I^2^ = 59%); thus, the fixed effect model could not be used. In contrast, analysis using a random-effects model revealed no significant heterogeneity [standardized mean difference (SMD) = −0.13; 95% CI, −0.31 to 0.04; *p* = 0.14] (Figure 2).

##### Activities of Daily Living

**Figure 3:**
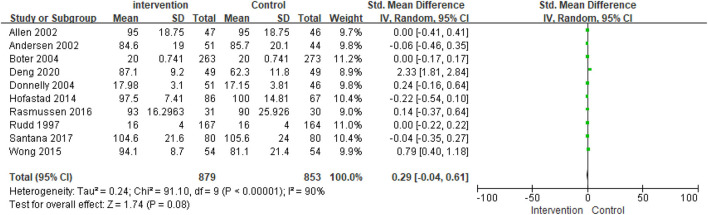
Comparison ESD care vs. usual care; outcome II: activities of daily living (ADL).

The ADL score was reported in 10 studies. The tool used to measure ADL (Barthel index, Modified Barthel index, Functional independent measure) was different in each of the studies; therefore, the scores were standardized and compared. First, a meta-analysis of ADL using the fixed effect model led to significant heterogeneity among studies (I^2^ = 90%; *p* < 0.000001). Thus, the fixed effect model could not be used. A meta-analysis of ADL using the random-effects model revealed no significant heterogeneity among studies (SMD = 0.29; 95% CI, −0.04 to −0.61; *p* = 0.08) (Figure 3).

##### mRS

**Figure 4:**
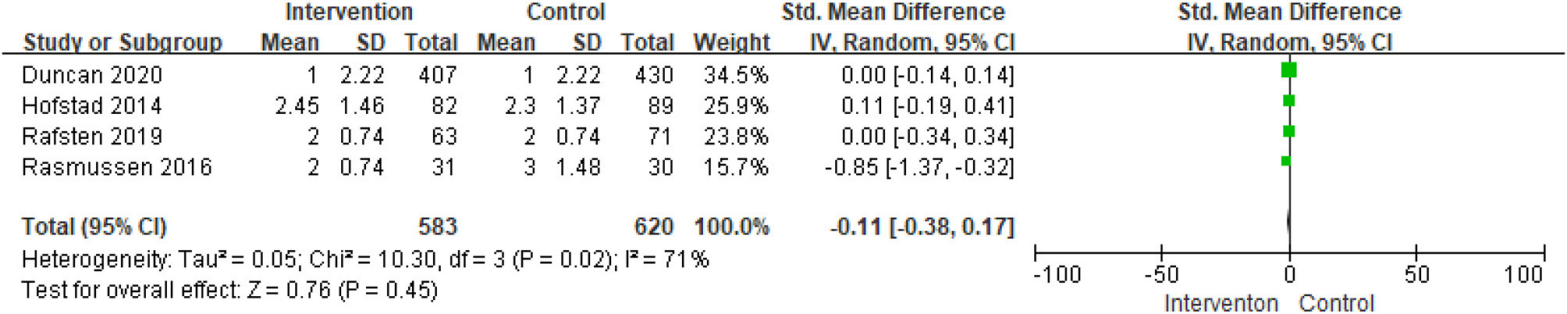
Comparison ESD care vs. usual care; outcome III: modified Rankin scale (mRS).

The mRS score was reported in four studies. Analysis of the mRS scores using the fixed effect model led to significant heterogeneity among studies (I^2^ = 71%, *p* = 0.02). Thus, the fixed effect model could not be used. A meta-analysis of mRS scores using the random-effects model revealed no significant heterogeneity among studies (SMD = −0.11; 95% CI, −0.38 to 0.17; *p* = 0.45) (Figure 4).

##### Death

**Figure 5:**
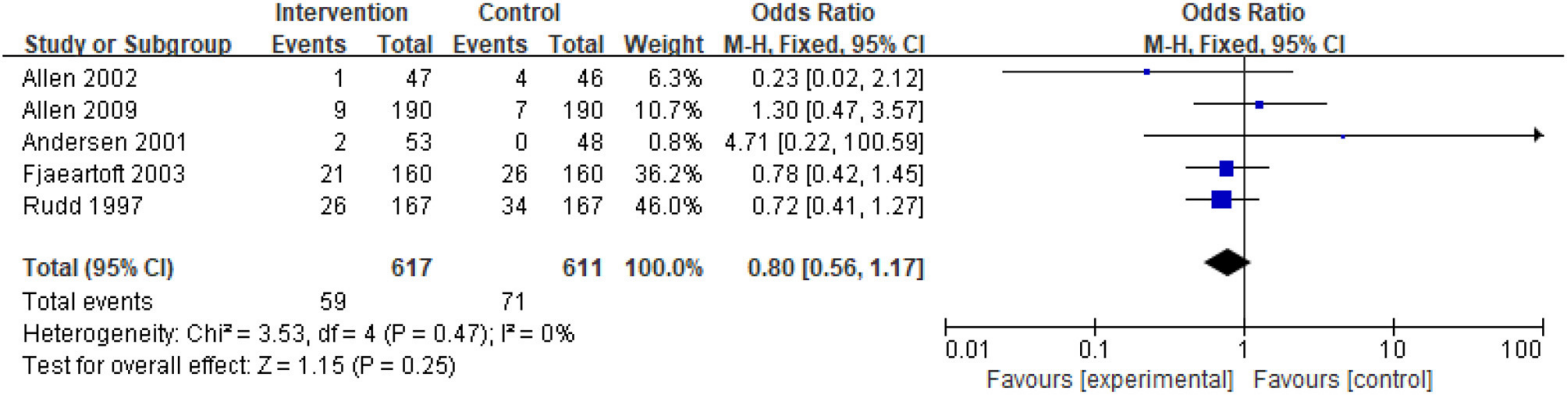
Comparison ESD care vs. usual care; outcome IV: death.

Death was reported in five studies. A meta-analysis of death using the fixed effect model revealed no significant heterogeneity among studies (I^2^ = 0%). Thus, the fixed effect model was used. However, the effect size was not significant [odds ratio (OR) = 0.80; 95% CI, 0.56–1.17; *p* = 0.25] (Figure 5).

##### Caregiver Strain Index

**Figure 6:**
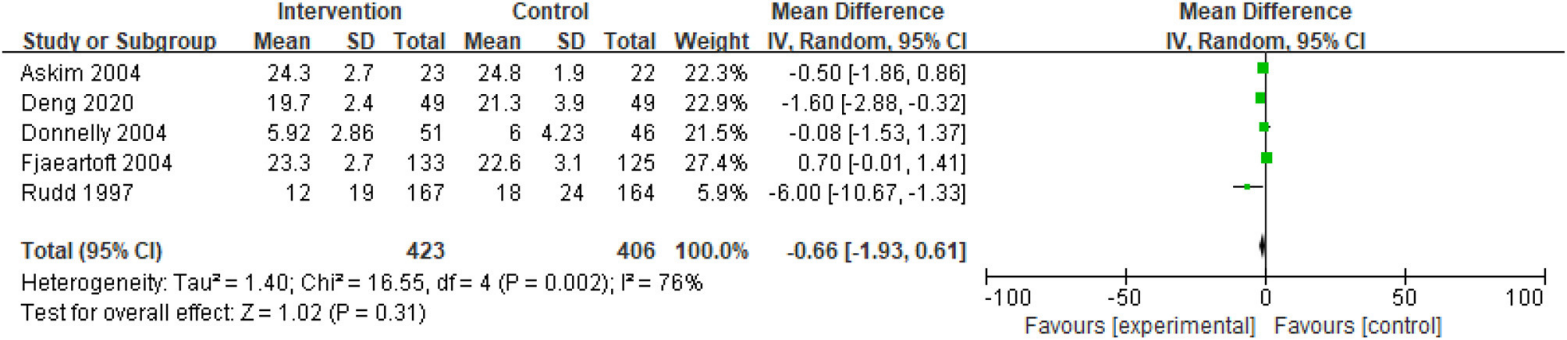
Comparison ESD care vs. usual care; outcome 5: Caregiver Strain Index (CSI).

Care burden was reported in five studies. A meta-analysis of care burden using the fixed effect model led to significant heterogeneity among studies (I^2^ = 76%). Thus, the fixed effect model could not be used. A meta-analysis of care burden using the random-effects model revealed no significant heterogeneity among studies (SMD = −0.66; 95% CI, −1.93 to 0.61; *p* = 0.31) (Figure 6).

##### RoB

**Figure 7:**
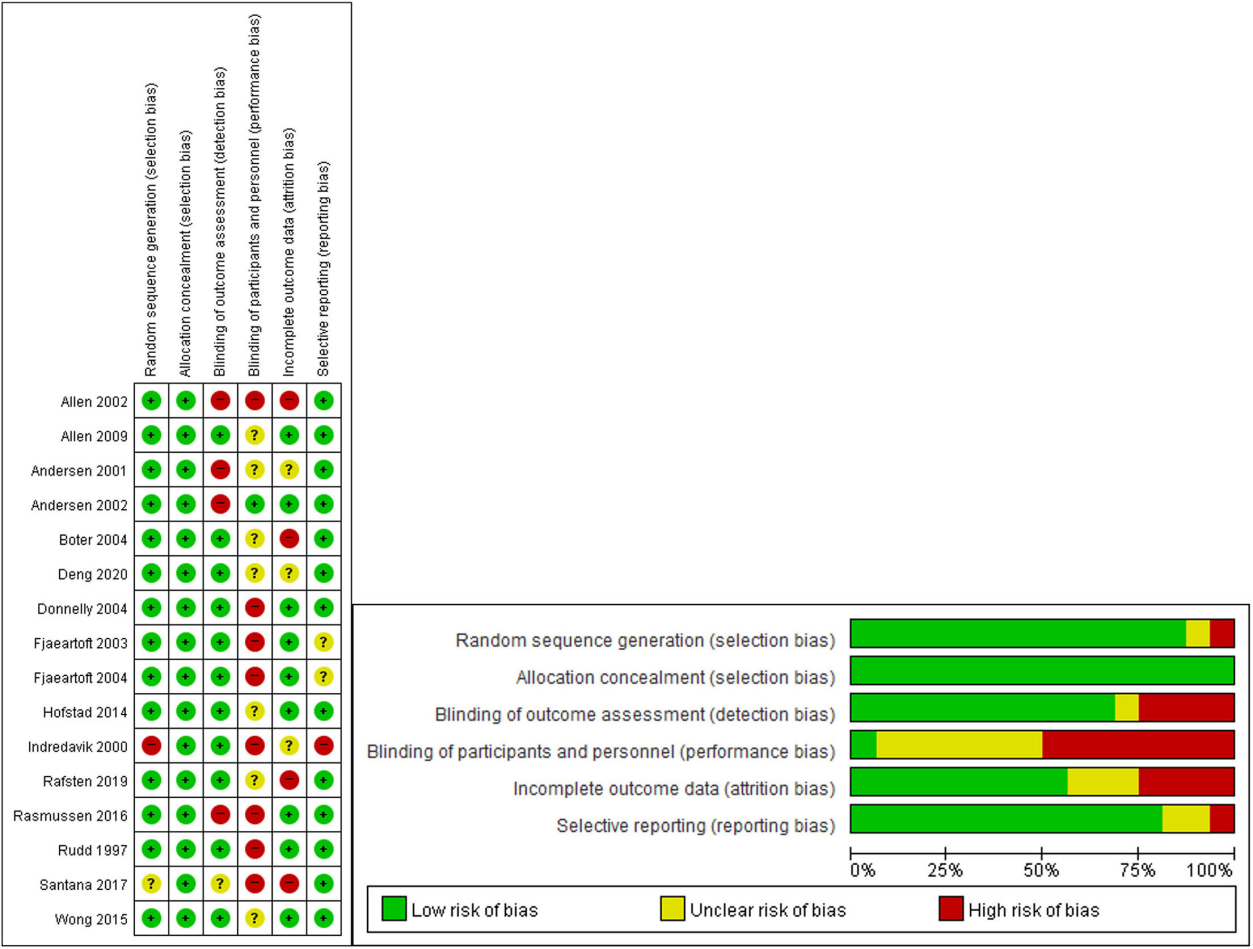
“Risk of bias” graph: review authors' judgments about each risk of bias item presented as percentages across included studies. “Risk of bias” summary: review authors' judgments about each risk of bias item for each included study.

There were some studies that revealed a high RoB in terms of performance bias, detection bias, and attrition bias. In contrast, the RoB was low for the selection bias and reporting bias. In particular, most studies revealed high or unclear RoB in performance bias (Figure 7).

##### Publication Bias

**Figure 8:**
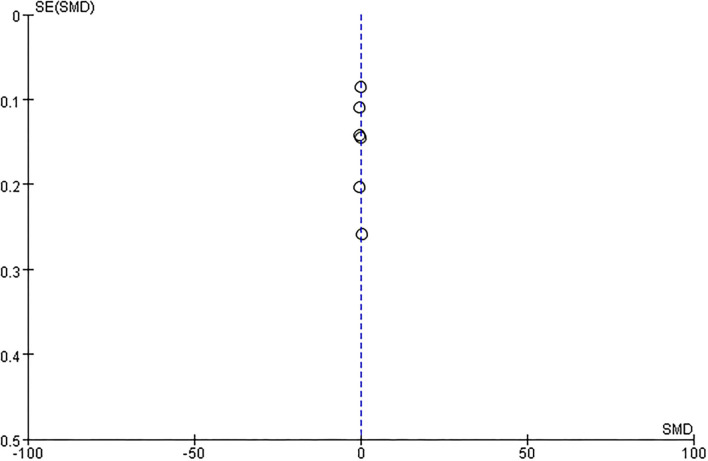
Funnel plot of comparison: ESD care vs. usual care, outcome: length of hospital stay.

**Figure 9:**
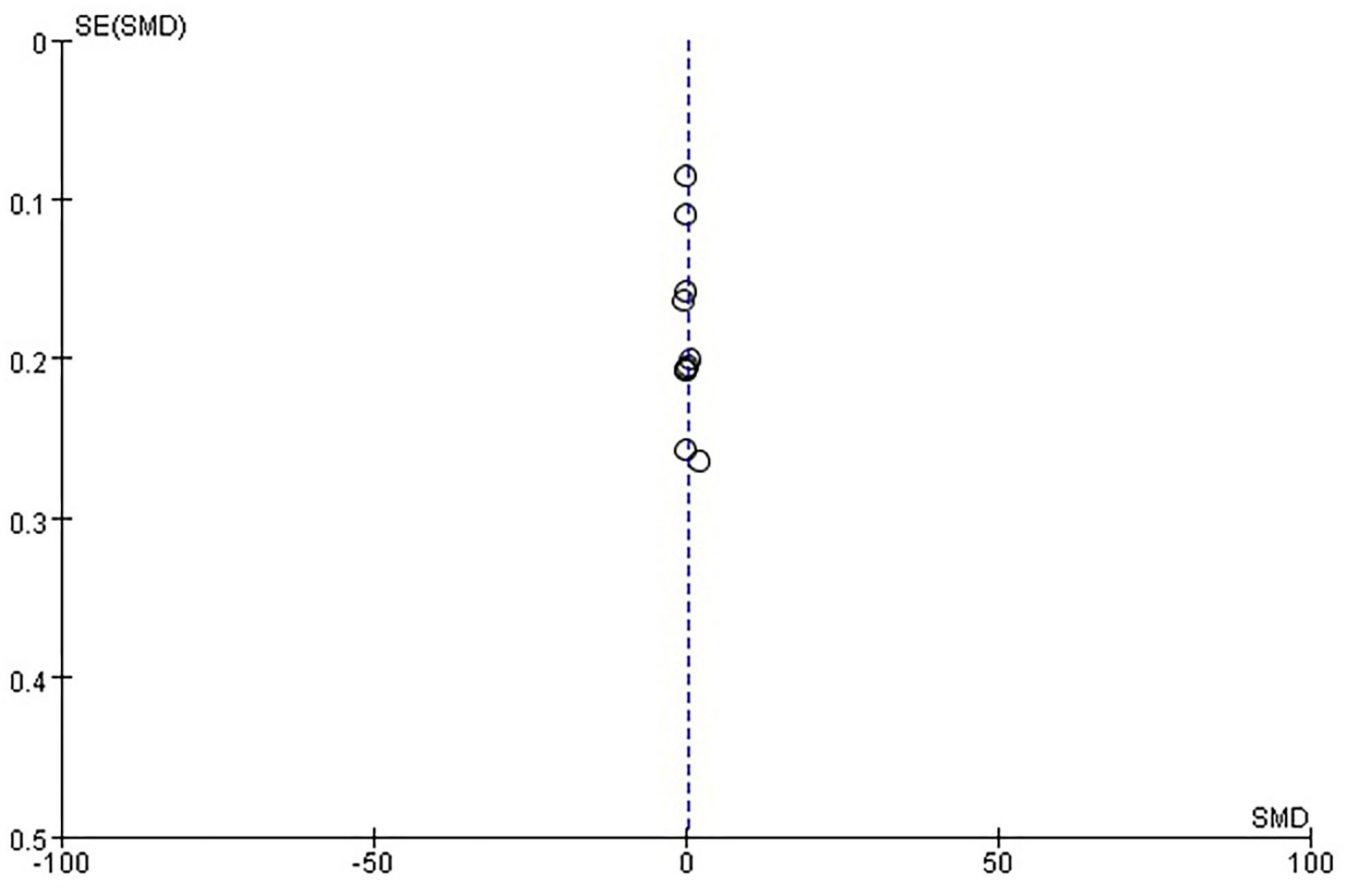
Funnel plot of comparison: ESD care vs. usual care, outcome: activities of daily living (ADL).

**Figure 10:**
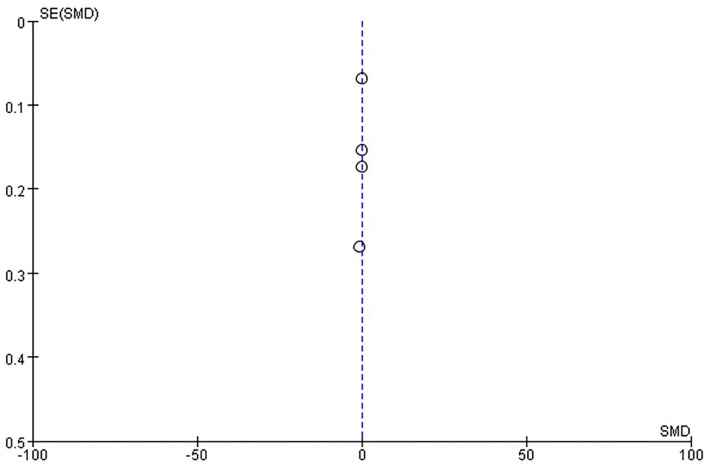
Funnel plot of comparison: ESD care vs. usual care, outcome: modified Rankin scale (mRS).

**Figure 11:**
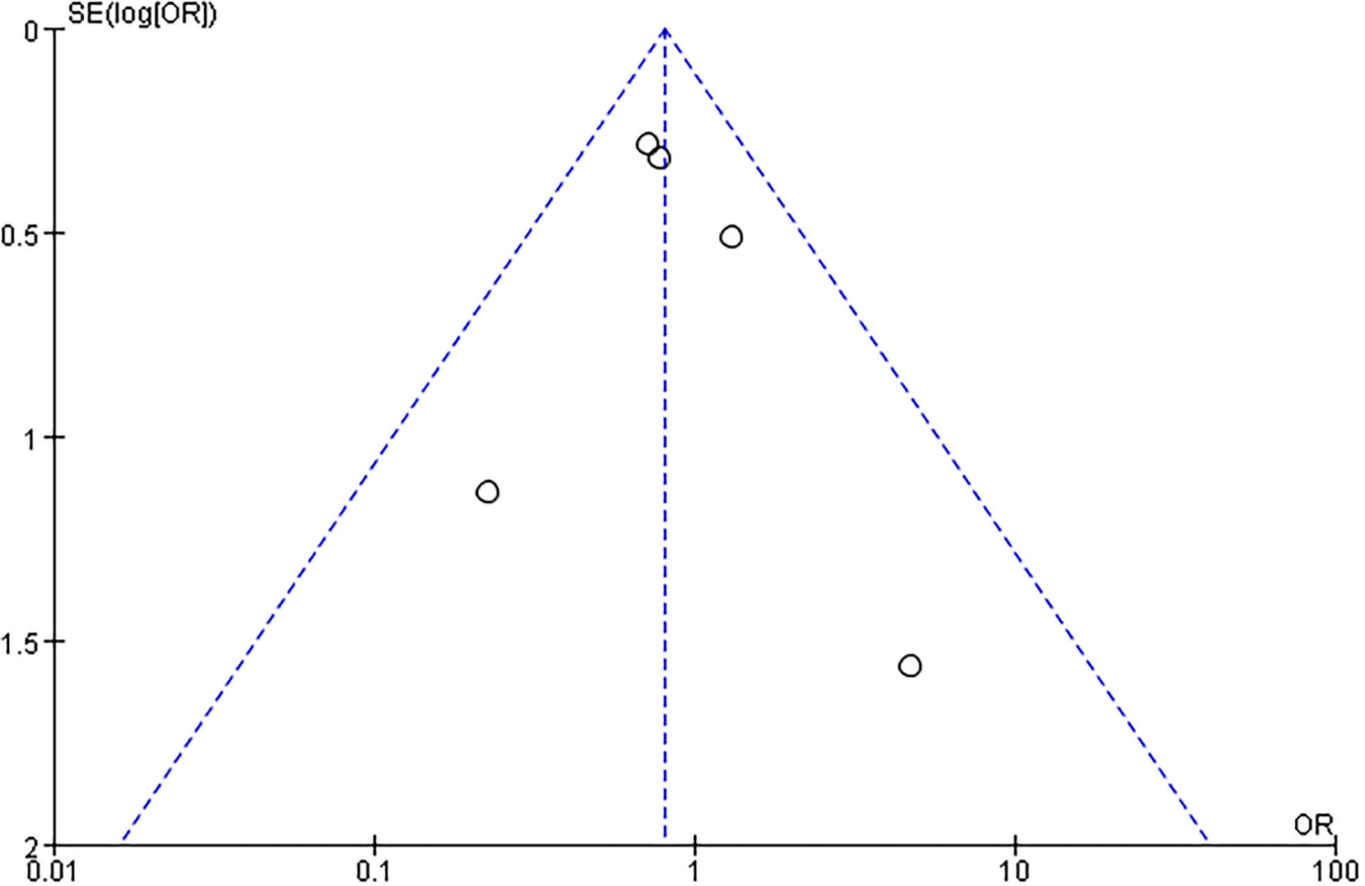
Funnel plot of comparison: ESD care vs. usual care, outcome: death.

There was no evidence of funnel plot asymmetry for length of hospital stay (Figure 8), ADL (Figure 9), mRS (Figure 10), and death (Figure 11). For the CSI, it was impossible to examine small study bias due to the small number of studies reporting cardiac events.

## Discussion

In this study, we systematically reviewed articles on ESD to assess the effects of ESD or TC on ADL, residual symptoms, quality of life, mortality, length of hospital stay, and caregiver burden. A total of 15,118 related studies were searched on three databases. The titles and abstracts were analyzed, and studies that were searched multiple times were excluded. Finally, this systematic literature review examined 20 studies (19 RCTs and 1 RPT). Among these 20 studies, two studies that followed up the participants for more than 5 years and a subgroup study were excluded, and 17 studies were finally included in the meta-analysis. In terms of location, 85, 10, and 5% of the 20 studies were conducted in Europe, Asia, and North America, respectively, demonstrating that ESD is mainly limited to western countries. However, recent studies on ESD have been reported in China, suggesting that ESD programs are also being implemented in Asian countries.

Hempler et al. conducted a systematic literature review of studies on transition management in Germany. A total of 18 studies were included in the literature review; however, there were no high-quality studies on standardized transition management systems, and the literature review study suggested that standardized discharge management services, including ESD programs, are needed in Germany. This finding is consistent with the results of our study, wherein studies conducted in Germany were not included in the meta-analysis. However, countries other than Germany are attempting various types of services, and the services are also gradually being offered in Asian countries, suggesting the need for these services in Korea. Unlike the literature review study conducted in Germany (29), our study is meaningful because two researchers independently selected and evaluated the studies for meta-analysis.

In 2017, Langhorne et al. conducted a literature search similar to our study and performed a systematic literature review and meta-analysis of 17 RCTs that included 2,422 patients. In that study, ESD reduced the length of hospital stay by ~6 days and might have reduced long-term functional dependence. In our study, there was a small number of cases in which ESD led to significant differences in the outcomes. This may be attributed to the homogeneous standard of interventions, excluding patient-led, family-led, and tele-rehabilitation, unlike those used in Cochrane's study, which deliberately set a broad criteria for interventions. Such differences led to exclusion of various studies, which might have led to a reduced number of cases in which ESD significantly improved the study outcomes (6).

We observed no significant differences in ADL between the TC methods, including ESD and conventional treatment. However, several points must be considered in the interpretation of this finding. As shown in the previous meta-analysis (6), ESD was mainly provided to high-level performing stroke patients. Thus, the ADL index included in our analysis might not accurately reflect the ADL functional status of patients due to the ceiling effects. Therefore, future studies must evaluate ADL using indicators that can evaluate high-level functions.

This systematic literature review and meta-analysis is meaningful because the most recent study (9) on TC has been included, and the types of services provided included ESD and TC in the literature search and selection.

However, this study also has several limitations. First, the definition of the interventions was unclear in each study. Thus, the type, duration, and period of intervention, and ESD team members were not completely identical. Second, most studies included in the meta-analysis were blinded to the assessor; however, the study participants could not be blinded. Thus, double-blinded studies could not be included. Third, pragmatic trials reflect realistic medical settings and could be as scientifically essential as an RCT, but it still needs a control group to be able to demonstrate an effect of the intervention. Lastly, we chose a particular set of trials relevant to the Republic of Korea. Therefore, caution is needed when interpreting the results of this study. We considered that TC programs should be a part of the ESD service in our country.

This study systematically investigated the effects of ESD and TC on medical use, function, mortality, and caregiver burden in stroke patients. We did not find a large effect size for the use of TC and ESD. When implementing the TC and ESD model from western to Asian countries, services should be prepared and implemented in accordance with national medical rehabilitation pathways for cerebrovascular disease.

## Data Availability Statement

The original contributions presented in the study are included in the article/Supplementary Material, further inquiries can be directed to the corresponding author/s.

## Author Contributions

SJ and MS contributed to the conception or design of the work. MJ, WK, Y-IS, and S-HK contributed to the data acquisition. IK, BC, YJ, and WC especially did statistical analysis and interpreted result of data for the work. All authors participated in drafting the work or revising it critically for important intellectual content, gave final approval of the version to be published, and agreed to be accountable for all aspects of the work in ensuring that questions related to the accuracy or integrity of any part of the work are appropriately investigated and resolved.

## Funding

This work was supported by the Research Program funded by the Korea National Institute of Health (Grant Number: 2020ER630601).

## Conflict of Interest

The authors declare that the research was conducted in the absence of any commercial or financial relationships that could be construed as a potential conflict of interest.

## Publisher's Note

All claims expressed in this article are solely those of the authors and do not necessarily represent those of their affiliated organizations, or those of the publisher, the editors and the reviewers. Any product that may be evaluated in this article, or claim that may be made by its manufacturer, is not guaranteed or endorsed by the publisher.
