# Supplementary material for: Early Supported Discharge and Transitional Care Management After Stroke: A Systematic Review and Meta-Analysis
Source: Front Neurol. 2022 Mar 15;13:755316. doi: 10.3389/fneur.2022.755316 (PMC8965290; doi:10.3389/fneur.2022.755316)
Supplement: Supplementary file 1 [file Table_1.docx]

Supplementary Table 1. Included variables to be analyzed

| **category** | **variables** |
| --- | --- |
| General characteristics | - Author and Year of publication - Study design - Country - Follow-up duration - Inclusion criteria - Intervention Type |
| Clinical characteristics | - Sample size - Disease category - Intervention Group: place, elements, conductor, duration, frequency, intensity - Control Group: place, elements, conductor |
| Clinical efficacy | - Primary outcome: Length of stay - Secondary outcome: ADL, mRS, Death, Quality of life, Readmission, Care burden |
