# Supplementary material for: Early Supported Discharge and Transitional Care Management After Stroke: A Systematic Review and Meta-Analysis
Source: Front Neurol. 2022 Mar 15;13:755316. doi: 10.3389/fneur.2022.755316 (PMC8965290; doi:10.3389/fneur.2022.755316)
Supplement: Supplementary file 2 [file Table_2.docx]

Supplementary Table 2. Guideline for Risk of Bias

| **Domain** | **Items** | **Result** |
| --- | --- | --- |
| Selection bias | Sequence generation | Low/  Unclear/  High |
|  | Allocation concealment |  |
| Performance bias | Blinding of participants, personnel |  |
| Detection bias | Blinding of Results assessment |  |
| Attrition bias | Incomplete Results data |  |
| Reporting bias) | Selective Results reporting |  |
