# Supplementary material for: Early Supported Discharge and Transitional Care Management After Stroke: A Systematic Review and Meta-Analysis
Source: Front Neurol. 2022 Mar 15;13:755316. doi: 10.3389/fneur.2022.755316 (PMC8965290; doi:10.3389/fneur.2022.755316)
Supplement: Supplementary file 3 [file Table_3.docx]

Supplementary table 3. characteristics of Included studies

| No. | Study Design | First Author | Year of publication | Nation | Population | Intervention Type | Intervention Group | | | Control Group | | F/U Duration | Results |
| --- | --- | --- | --- | --- | --- | --- | --- | --- | --- | --- | --- | --- | --- |
|  |  |  |  |  |  |  | Intervention Characteristic | | N | Characteristic | N |  |  |
| 1 | RCT | Allen | 2002 | Denmark | Patients diagnosed with ischemic stroke | I | · PSC (poststroke consultation) team member : geriatrician, the APN-CM(advanced practice nurse care manager), clinical nurse specialist, neurologist, pharmacist, dietitian, social worker  · one time home visit, consultation, treatment plan for prevention | | 47 | usual care | 46 | 3 months | · BI  · Death  · Readmission for stroke |
| 2 | RCT | Allen | 2009 | Denmark | Ischemic stroke | I | · in-home assessment by APN-CM within 1 week of discharge.  · home visits by a physical therapist  · contact by telephone once a week for the 1st month postdischarge, then once a month until the end of the study | | 190 | usual postdischarge care | 190 | 6 months | · Death  · QoL |
| 3 | RCT | Andersen | 2000 | Denmark | Stroke patients | I | the physician intervention consisted of three 1-hour home visit  (at 2, 6 and 12 weeks after discharge) | | 54 | standard aftercare | 48 | 6 months | · Readmission  · Readmission >1  · Readmission due to stroke-related condition  · Death |
|  |  |  |  |  |  | I | (1) instruction and reeducation by the hospital physiotherapist during a 6- week period after discharge.  (2) the visits took place in the patient’s home  (3) each visit lasted ~1 hour  (4) the average number of visits per patient was 2.9 (range 1to 8) | | 53 |  |  |  |  |
| 4 | RCT | Andersen | 2002 | Denmark | Stroke patients | I | the physician intervention consisted of three 1-hour home visit(at 2, 6 and 12 weeks after discharge) | | 51 | standard aftercare | 43 | 6 months | · BI |
|  |  |  |  |  |  | I | (1) instruction and reeducation by the hospital physiotherapist during a 6- week period after discharge.  (2) the visits took place in the patient’s home  (3) each visit lasted ~1 hour  (4) the average number of visits per patient was 2.9 (range 1to 8) | | 44 |  |  |  |  |
| 5 | RCT | Askim | 2004 | Norway | Patients admitted to the Stroke Unit | II | · Stroke Unit combined with a home-based programme of follow-up care co-ordinated by a mobile stroke team that offers ESD and works in close co-operation with the primary health care system during the first 4 weeks after discharge, 30~45 min radius from hospital  · The mobile team was based within the stroke unit and consisted of a nurse, a physiotherapist, an occupational therapist and the consulting service of a physician | | 31 | acute care in a stroke unit | 31 | 6/26/52 weeks | · mRS≤2(%)  · BI  · CSI  · CSI |
| 6 | RCT | Boter | 2004 | Netherlands | Stroke patients | I | 3 telephone calls(1 to 4, 4 to 8, 18 to 24 weels after discharge) and 1 home visit within 5 months after discharge by a stroke nurse | | 231 | standard care only | 255 | 6 months | · Readmission  · BI  · mRS  · CSI |
| 7 | RCT | Deng | 2020 | China | Patients with acute cerebral hemorrhage or cerebral infarction | II | (1) ongoing stroke rehabilitation performed by rehabilitation therapist  (2) medication reconciliation performed by general practitioners and nurses  (3) self-management education regarding risk factors control and stroke warning signs  · The scheduled treatment for the intervention group was eight weeks and maximally 2 hours per day | | 49 | usual postdischarge care was administered by the staff from community healthcare stations. | 49 | 4/8 weeks | · MBI  · CSI |
| 8 | RCT | Donelly | 2004 | UK | Stroke patinets | II | · The community-based multidisciplinary stroke team (CST) service  · team member: 0.33 coordinator, 1 occupational therapist, 1.5 physiotherapists, 1 speech and language therapist, and 2 rehabilitation assistants  · On average the number of home visits (each lasting 45 minutes) over a 3-month period was 2.5 per week. | | 51 | usual inpatient rehabilitation | 46 | 12 months | · BI,  · EuroQol,  · CSI |
| 9 | RPT | Duncan | 2020 | USA | Patients with ischemic stroke, hemorrhagic stroke, or TIA | I | telephone follow-up within 2 business days of hospital discharge and a clinic visit targeted to occur 7 to 14 days post-discharge | | 1529 | standard of postacute care | 1680 | 3 months | · mRS |
| 10 | RCT | Fjæartoft | 2003 | Norway | Acute stroke patients | II | · ESUS; extended stroke unit service  · comprehensive follow-up stroke service organized by a mobile team. · This hospital-based team consisted of a physiotherapist, an occupational therapist, a nurse, and the consulting service of a stroke physician  · home visit: within 3 to 5 days after admission to the SU | | 160 | · OSUS; ordinary stroke unit service | 160 | 52 weeks | · RS≤2(%)  · BI≥95(%)  · Death (%) |
| 11 | RCT | Fjæartoft | 2004 | Norway | Acute stroke patients | II | · ESUS; extended stroke unit service  · comprehensive follow-up stroke service organized by a mobile team. · This hospital-based team consisted of a physiotherapist, an occupational therapist, a nurse, and the consulting service of a stroke physician  · home visit: within 3 to 5 days after admission to the SU | | 133 | · OSUS; ordinary stroke unit service | 125 | 52 weeks | · CSI |
| 12 | RCT | Fjæartoft | 2011 | Norway | Acute stroke patients | II | The mobile team consisted of a physiotherapist, an occupational therapist, a nurse, and the part-time service of a physician. | | 160 | · OSUS; ordinary stroke unit service | 160 | 5 years | · Death (%)  · mRS≤2 (%)  · BI≥95 (%) |
| 13 | RCT | Hofstad | 2014 | Norway | Patients with acute stroke | II | Day unit: treatment in a community day unit | (1) rehabilitative treatment by a multi-disciplinary community health team, consisting of a nurse, a physiotherapist and an occupational therapist  (2) treatment period was 5 weeks and maximally 4 hours per day 5 days a week | 103 | institutional stay if necessary and/or physiotherapy as needed in the municipality | 99 | 3/6 months | · mRS  · BI  · Length of Stay |
|  |  |  |  |  |  | II | Hom based: home-visits from the community health team. |  | 104 |  |  |  |  |
| 14 | RCT | Indredavik | 2000 | Norway | Patients with acute stroke | II | · ESUS; extended stroke unit service  · comprehensive follow-up stroke service organized by a mobile team. · This hospital-based team consisted of a physiotherapist, an occupational therapist, a nurse, and the consulting service of a stroke physician | | 121 | · OSUS; ordinary stroke unit service | 122 | 6/26 weeks | · Death (%)  · BI≥95 (%)  · RS≤2 (%) |
| 15 | RCT | Rafsten | 2019 | Sweden | Patients admitted to stroke care units | II | (1) 2-4 visits per week by the physiotherapist and/or occupational therapist  (2) 1-2 visits by the stroke nurse  (3) the maximum length was 4 weeks after discharge | | 69 | usual routine | 71 | 3/12 months | · BI  · RS |
| 16 | RCT | Rasmussen | 2016 | Denmark | Acute stroke patients | II | (1) before discharge  · representatives of the team drove the inpatient home one to three times per week.  (2) after discharge  · continued rehabilitation training at home one to five days per week by the multidisciplinary team. | | 31 | conventional care | 30 | 3 months | · LOS  ·RS  · BI  · EQ-5D |
| 17 | RCT | Rudd | 1997 | UK | Stroke patients | II | (1) planned course of domiciliary physiotherapy, occupational therapy, and speech therapy, with visits as frequently as considered appropriate (maximum one daily visit from each therapist).  (2) Patients received care from the team for a maximum of 3 months. | | 167 | conventional care | 164 | 1 year | · BI  · Careiver strain Index  · Death  · Readmission  · LOS from randomisation, |
| 18 | RCT | Santana | 2017 | Portugal | Stroke patients | II | · early home-supported discharge (EHSD) started during their stay at the stroke unit  · 2 physiotherapists, 2 occupational therapists and a psychologist.  · The EHSD team worked with patients to provide approximately 8 home-based training sessions for a maximum of one month | | 95 | standard care | 95 | 2/6 months | · FIM |
| 19 | RCT | Thorsén | 2005 | Sweden | Stroke patients | II | · home rehabilitation: The mean duration of the intervention program was 14 weeks; the mean number of home visits was 12  · An outreach team of occupational therapists, physiotherapists, and a speech-and-language pathologist provided ESD service | | 42 | conventional rehabilitation | 41 | 5 years | · BI |
| 20 | RCT | Wong | 2015 | Hong Kong | Stroke patients | I | · Pre-discharge – entrance family meeting  · Week 1 – home visit on day 2–4, telephone follow-up on day 7  · Week 2 – home visit on day 2–4, telephone follow-up on day 7  · Week 3 – home visit on day 2–4, telephone follow-up on day 7  · Week 4 – home visit on day 2–4, exit family meeting | | 54 | routine hospital-based physical training programme | 54 | 4/8 weeks | · WHOQOL-SRPB-HK  · MBI |
| RCT, Randomized Controlled Trial; RPT, Randomized Pragmatic Trial; BI, Barthel Index; MBI, Modified Barthel Index; RS, Rankin Scale; mRS, modified Rankin Scale; CSI, Caregiver Strain Index; FIM, Functional Independence Measure; LOS. Length of Stay; QoL, Quality of Life; EQ-5D, EuroQoL-5D; WHOQOL-SRPB-HK, World Health Organization Quality of Life -Spirituality, Religiousness, and Personal Beliefs-Hong Kong | | | | | | | | | | | | | |
