# Supplementary material for: Early Supported Discharge and Transitional Care Management After Stroke: A Systematic Review and Meta-Analysis
Source: Front Neurol. 2022 Mar 15;13:755316. doi: 10.3389/fneur.2022.755316 (PMC8965290; doi:10.3389/fneur.2022.755316)
Supplement: Supplementary file 4 [file Table_4.docx]

Supplementary table 4. Comparison of various clinical outcomes according to included Studies

| No. | Intervention Type | Variables | Intervention Group | Control Group |  |  |
| --- | --- | --- | --- | --- | --- | --- |
|  |  |  | Intervention Group (n=47) | Control Group (n=46) | Range | difference |
| 1 | type I | 3 months after discharge | | | | |
|  |  | BI (mean) | 95 | 95 | 25-100 | 0 |
|  |  | Death (%) | 2 | 9 |  |  |
|  |  | readmission (%) | 2 | 0 |  |  |
|  |  |  | Intervention Group (n=190) | Control Group (n=190) | difference (SD) | 95% CI |
| 2 | type II | 6 months after discharge | | | | |
|  |  | Death (%) | 4.5 | 3.5 | 2.2 (0.01) | (╶3.1, 5.3) |
|  |  | average QOL | 196 | 199 | ╶2 (╶0.07) | (╶9, 5) |
|  |  |  | Intervention Group1 (n=54) | Intervention Group2 (n=53) | Control Group (n=48) | p-value |
| 3 | type I | within 6 months after discharge | | | | |
|  |  | Readmission; n (%) | 14 (26) | 18 (34) | 21 (44) | 0.028 |
|  |  | Readmission >1; n (%) | 4 (7) | 2 (4) | 7 (15) | 0.14 |
|  |  | stroke-related readmission; n (%) | 7 (13) | 11 (21) | 15 (31) | 0.079 |
|  |  | Death | 1 | 2 | 0 |  |
|  |  |  | Intervention Group1 (n=51) | Intervention Group2 (n=44) | Control Group (n=43) | p-value |
| 4 | type I | 6 months after discharge | | | | |
|  |  | BI; mean ± SD | 84.6(19.0 | 85.7±20.1 | 77.5±25.9 | 0.165 |
|  |  |  |  |  |  |  |
|  |  |  | Intervention Group (n=31) | Control Group(n=31) | p-value | 95% CI |
| 5 | type II | 6 weeks after stroke onset | | | | |
|  |  | mRS ≤ 2 (%) | 16 (51.6) | 16 (51.6) | 1 | ╶ 0.26 to 0.26 |
|  |  | BI ≥ 95 (%) | 13 (41.9) | 14 (45.2) | 1 | ╶ 0.28 to 0.22 |
|  |  | BI, mean (SD) | 75.2 (30.6) | 74.0 (31.2) | 0.769 | ╶ 15.1 to 17.5 |
|  |  | CSI | (n=29) | (n=29) |  |  |
|  |  | median (IQR) | 26.0 (23.5-26.0) | 24.0 (23.0-26.0) | 0.107 |  |
|  |  | mean (SD) | 24.5 (2.3) | 23.5 (2.4) |  |  |
|  |  | 26 weeks after stroke onset | | | | |
|  |  | mRS ≤ 2 (%) (n) | 13 (41.9) (31) | 16 (51.6) (31) | 0.611 | ╶ 0.35 to 0.16 |
|  |  | BI ≥ 95 (%) (n) | 11 (35.5) (31) | 14 (45.2) (31) | 0.605 | ╶ 0.34 to 0.15 |
|  |  | BI, mean (SD) (n) | 78.0 (32.9) (23) | 77.7 (27.6) (28) | 0.914 | ╶ 20.0 to 14.7 |
|  |  | CSI | (n=22) | (n=23) |  |  |
|  |  | median (IQR) | 26.0 (21.8-26.0) | 26.0 (24.0-26.0) | 0.429 |  |
|  |  | mean (SD) | 24.2 (2.5) | 25.0 (1.6) |  |  |
|  |  | 52 weeks after stroke onset | | | | |
|  |  | mRS ≤ 2 (%) (n) | 12 (38.7) (31) | 16 (51.6) (31) | 0.444 | ╶ 0. 37 to 0.13 |
|  |  | BI ≥ 95 (%) (n) | 11 (35.5) (31) | 15 (48.4) (31) | 0.44 | ╶ 0. 37 to 0.12 |
|  |  | BI, mean (SD) (n) | 71.7 (34.7) (23) | 790 (28.7) (25) | 0.45 | ╶ 25.9 to 11.4 |
|  |  | CSI | (n=23) | (n=22) |  |  |
|  |  | median (IQR) | 26.0 (23.0-26.0) | 26.0 (24.0-26.0) | 0.832 |  |
|  |  | mean (SD) | 24.3 (2.7) | 24.8 (1.9) |  |  |
|  |  |  | Intervention Group (n=231) | Control Group (n=255) | 95% CI |  |
| 6 | type I | 6 months after discharge | | | | |
|  |  | readmission§ | 35/215 (16%) | 28/243 (12%) | 1.41 (0.89 to 2.24) |  |
|  |  | BI; median (IQR) | 20 (20 to 20) | 20 (19 to 20) | 0 (0 to 0) |  |
|  |  | mRS; median (IQR) | 1 (1 to 2) | 1 (1 to 2) | 0 (╶ 0.32 to 0.39) |  |
|  |  | CSI; median (IQR) (n) | 3 (0 to 5) (n=162) | 3 (1 to 6) (n=230) | 0 (- 1.58 to 3.44) |  |
|  |  |  | Intervention Group (n=49) | Control Group (n=49) | p-value | F |
| 7 | type II | 4 weeks after discharge | | | | |
|  |  | MBI, mean (SD) | 87.1 (9.2) | 62.3 (11.8) | 0* | 20.09 |
|  |  | CSI, mean (SD) | 23.6 (1.2) | 24.1 (0.9) | 0.208 | 6.35 |
|  |  | 8 weeks after discharge | | | | |
|  |  | MBI, mean (SD) | 92.5 (6.7) | 77.1 (1.3) | 0* | 16.78 |
|  |  | CSI, mean (SD) | 19.7 (2.4) | 21.3 (3.9) | 0* | 8.78 |
|  |  |  | Intervention Group (n=51) | Control Group (n=46) | p-value | 95% CI |
| 8 | type II | 12 months after discharge | | | | |
|  |  | BI |  |  |  |  |
|  |  | mean (SD) | 17.98 (3.10) | 17015 (3.81) | 0.179 | ╶2.24 to 0.58 |
|  |  | median | 20 | 18 |  |  |
|  |  | range | 15 | 16 |  |  |
|  |  | EuroQol |  |  |  |  |
|  |  | mean (SD) | 66.36 (18.45) | 68.21 (20.31) | 0.604 | ╶6.20 to 9.90 |
|  |  | median | 70 | 70 |  |  |
|  |  | range | 100 | 100 |  |  |
|  |  | Carer Strain |  |  |  |  |
|  |  | mean (SD) | 5.92 (2.86) | 6.00 (4.23) | 0.927 | ╶2.14 to 2.30 |
|  |  | median | 6 | 5 |  |  |
|  |  | range | 12 | 12 |  |  |
|  |  |  | Intervention Group (n=1529) | Control Group (n=1680) | 95%CI |  |
| 9 | type I | 3 months after discharge | | | | |
|  |  | mRS, median (IQR) | 1 (0-3) | 1 (0-3) (1680) | 0.88 (0.77 to 0.01) |  |
|  |  |  | Intervention Group (n=160) | Control Group (n=160) | p-value |  |
| 10 | type II | 52 weeks after stroke onset | | | | |
|  |  | RS ≤ 2 (%) (n) | 56.3 (90) | 45.0 (72) | 0.044 |  |
|  |  | BI ≥ 95 (%) (n) | 52.5 (84) | 46.3 (74) | 0.0264 |  |
|  |  | Dead (%) (n) | 13.1 (21) | 16.3 (26) | 0.429 |  |
|  |  |  | Intervention Group (n=133) | Control Group (n=125) | p-value |  |
| 11 | type II | 52 weeks after stroke onset | | | | |
|  |  | CSI |  |  |  |  |
|  |  | Mean (SD) | 23.3 (2.7) | 22.6 (3.1) | 0.089 |  |
|  |  | Median (range) | 24.0 (15-26) | 23.0 (14-26) |  |  |
|  |  |  | Intervention Group (n=160) | Control Group (n=160) | p-value |  |
| 12 | type II | 5 years after stroke onset | | | | |
|  |  | Dead (%) (n) | 45.8 (71) | 51.0 (77) | 0.364 |  |
|  |  | mRS ≤ 2 (%) (n) | 34.8 (54) | 25.8 (43) | 0.213 |  |
|  |  | BI ≥ 95 (%) (n) | 48 (57.1) (84) | 38 (51.4) (74) | 0.285 |  |
|  |  |  | Intervention Group 1 | Intervention Group 2 | Control Group | p-value |
| 13 | type II | 3 months after stroke onset | | | | |
|  |  | mRS; mean (SD) (n) | 2.45 (1.46) (82) | 2.30 (1.37) (89) | 2.62 (1.40) (71) | 0.316 |
|  |  | BI;median (IQR) (n) | 100 (15) (78) | 97.5 (10) (86) | 100 (20) (67) | 0.976 |
|  |  | 6 months after stroke onset | | | | |
|  |  | mRS; mean (SD) (n) | 2.40 (1.53) (81) | 2.46 (1.45) (82) | 2.73 (1.52) (66) | 0.41 |
|  |  | BI;median (IQR) (n) | 100 (15) (76) | 100 (10) (77) | 100 (15) (60) | 0.977 |
|  |  | until 6 months after inclusion | | | | |
|  |  | Days in institution; mean (SEM) | (n=103) | (n=104) | (n=99) |  |
|  |  | SU | 11.3 (0.6) | 11.3 (0.7) | 11.6 (0.8) | 0.919 |
|  |  | DPMR | 7.9 (2.0) | 7.6 (1.9) | 6.5 (2.3) | 0.56 |
|  |  | municipal institution | 23.4 (24.6) | 21.5 (4.5) | 27.4 (4.5) | 0.111 |
|  |  |  | Intervention Group (n=121) | Control Group (n=122) | p-value |  |
| 14 | type II | 6 weeks after stroke onset | | | | |
|  |  | Dead (%) (n) (n=160) | 2.5 (4) | 4.4 (7) | 0.357 |  |
|  |  | BI ≥ 95 (%) (n) | 46.3 (56) | 34.4 (42) | 0.06 |  |
|  |  | RS ≤ 2 (%) (n) | 43.0 (52) | 31.2 (38) | 0.056 |  |
|  |  | 26 weeks after stroke onset | | | | |
|  |  | Dead (%) (n) (n=160) | 8.1 (13) | 9.4 (15) | 0.692 |  |
|  |  | BI ≥ 95 (%) (n) | 52.1 (63) | 38.5 (47) | 0.034* |  |
|  |  | RS ≤ 2 (%) (n) | 57.9 (70) | 40.2 (49) | 0.006* |  |
|  |  |  | Intervention Group | Control Group | p-value |  |
| 15 | type II | 3 months after stroke onset | | | | |
|  |  | BI, median (IQR) (n) | S (69) | 100 (90-100) (71) | 0.22 |  |
|  |  | mRS, median (IQR) (n) | 2 (1-2) (63) | 2 (2-3) (71) | ≤0.01* |  |
|  |  | 12 months after stroke onset | | | | |
|  |  | BI, median (IQR) (n) | 100 (90-100) (69) | 100 (85-100) (71) | 0.76 |  |
|  |  | mRS, median (IQR) (n) | 2 (1-3) (63) | 2 (1-3) (71) | 0.08 |  |
|  |  |  | Intervention Group (n=31) | Control Group (n=30) | p-value |  |
| 16 | type II | 3 months after stroke onset | | | | |
|  |  | LOS, days; median (IQR) | 18(16-21) | 16(12-21) |  |  |
|  |  | mRS; median (IQR) | *2 (2-3) | 3 (2-4) |  |  |
|  |  | BI; median (IQR) | 93 (78-100) | 90 (60-95) |  |  |
|  |  | EuroQol-5D; median (IQR) | *0.77 (0.66-0.79) | 0.66 (0.56-0.72) |  |  |
|  |  |  | Intervention Group (n=167) | Control Group (n=164) | p-value | 95% CI |
| 17 | type II | 1 year after stroke onset | | | | |
|  |  | BI |  |  |  |  |
|  |  | mean (SD) | 16 (4) | 16 (4) |  |  |
|  |  | median (range) | 18 (2-20) | 18 (3-20) | 0.3 | 0to1 |
|  |  | not assessed | 1 | 0 |  |  |
|  |  | Caregiver strain |  |  |  |  |
|  |  | mean (SD) | 5 (4) | 4 (3) |  |  |
|  |  | median (range) | 5 (0-12) | 3 (0-12) | 0.14 | 0 to 2 |
|  |  | not assessed | 61 | 67 |  |  |
|  |  | Dead by 12 months | 26 (16) | 34 (21) | 0.22 | (-13 to 3) |
|  |  | Readmitted to hospital | 44 (26) | 42 (26) | 0.2 | (-9 to 10) |
|  |  | > 12 months trial period | | | | |
|  |  | LOS from randomization |  |  |  |  |
|  |  | mean (SD) | 12 (19) | 18 (24) |  |  |
|  |  | median (range) | 6 (0-149) | 12 (0-236) | 0.0001* | (-6 to -2) |
|  |  | not known | 2 | 1 |  |  |
|  |  |  | Intervention Group | Control Group | p-value |  |
| 18 | type II | at 2^nd^ months | | | | |
|  |  | FIM; mean (SD) (min-max) (n) | 104.6 (21.6) (53-126) (80) | 105.6 (24.0) (20-126) (80) | 0.798 |  |
|  |  | at 6^th^ months | | | | |
|  |  | FIM; mean (SD) (min-max) (n) | 107.4 (19.9) (45-126) (74) | 106.6 (25.5) (18-126) (78) | 0.816 |  |
|  |  |  | Intervention Group (n=30) | Control Group (n=24) | p-value |  |
| 19 | type II | 5 years after stroke onset | | | | |
|  |  | independence in BI | 20 (67)† | 14 (58)† | 0.28 |  |
|  |  |  | Intervention Group (n=54) | Control Group (n=54) | p-value |  |
| 20 | type I | 4 weeks after inclusion | | | | |
|  |  | WHOQOL-SRPB-HK; mean (SD) | 12.5(3.1) | 9.8 (2.5) | 0.001** |  |
|  |  | MBI, mean (SD) | 90.0 (11.8) | 78.8 (20.9) | <0.001** |  |
|  |  | 8 weeks after inclusion | | | | |
|  |  | WHOQOL-SRPB-HK; mean (SD) | 12.1 (2.9) | 9.8 (2.7) | 0.002** |  |
|  |  | MBI, mean (SD) | 94.1 (8.7) | 81.1 (21.4) | <0.001** |  |
| RCT, Randomized Controlled Trial; RPT, Randomized Pragmatic Trial; BI, Barthel Index; MBI, Modified Barthel Index; RS, Rankin Scale; mRS, modified Rankin Scale; CSI, Caregiver Strain Index; FIM, Functional Independence Measure; LOS. Length of Stay; QoL, Quality of Life; EQ-5D, EuroQoL-5D; WHOQOL-SRPB-HK, World Health Organization Quality of Life -Spirituality, Religiousness, and Personal Beliefs-Hong Kong | | | | | | |
